# Entrepreneurs’ creativity, information technology adoption, and continuance intention: Mediation effects of perceived usefulness and ease of use and the moderation effect of entrepreneurial orientation

**DOI:** 10.1016/j.heliyon.2024.e25479

**Published:** 2024-02-02

**Authors:** Adin Kusumo Putro, Yoshi Takahashi

**Affiliations:** a Graduate School of Humanities and Social Sciences, Hiroshima University, Higashihiroshima, Japan; b Directorate General of Taxes, Ministry of Finance, Jakarta, Indonesia

**Keywords:** Entrepreneur, Information technology, Creativity, Entrepreneurial orientation, Technology acceptance model

## Abstract

Despite the potential of information technology (IT) to enhance business efficiency and productivity, many entrepreneurs still refuse to adopt it. Their low adoption rate of IT is often attributed to a lack of awareness of its potential benefits. Extending the technology acceptance model, this study aimed to investigate the effect of creativity mediated by perceived usefulness (PU) and perceived ease of use (PEOU) on the intention to adopt and continue using IT. Additionally, the study explores how entrepreneurial orientation (EO) functions as a contextual factor in the relationship between creativity, PU, and PEOU. In contrast to extant studies, EO is a better-suited variable for our study as it is more extensive. Data were collected through questionnaires distributed to individual entrepreneurial taxpayers registered in tax offices in the Special Region of Yogyakarta, Indonesia. The study employed not only a quasi-experimental method with coarsened exact matching to reduce the degree of model dependence, inefficiency, and bias but also a causal mediation analysis to explore the proposed relationships. The study focused on the adoption of DJP Online, an e-tax service provided by the Directorate General of Taxes as the Indonesian tax authority, and the e-marketplace. In contrast with the factors identified in extant studies, we explain that the variance in PU is attributed to a greater extent to creativity. Similarly, we find that creativity explains a larger portion of the variance in PEOU than that found in extant studies. Our findings thoroughly explain how creativity influences entrepreneurs’ willingness to adopt and continue using new technologies. Other studies could employ a more diverse and representative sample to extend these findings.

## 1 Introduction

The utilization of information technology (IT) has transformed how entrepreneurs conduct diverse business operations [1,2]. It can significantly benefit them by automating routine tasks and processes, improving business efficiency, enhancing productivity, reducing transaction costs, improving business performance, and providing information transparency. An e-marketplace and e-tax service are two such examples. By automating tasks and processes such as tax preparation and e-filing, entrepreneurs can reduce the time and effort required to complete them, allowing them to focus on more strategic activities [3,4]. This can help improve business efficiency and reduce the risks of errors while ensuring compliance with tax regulations in the case of an e-tax service [4]. IT can not only enhance productivity by providing better access to information and facilitating communication but also help entrepreneurs better track their business performance, make better decisions, and serve as a catalyst for fostering innovation [[4], [5], [6]].

Despite these benefits, the IT implementation rate among entrepreneurs has remained relatively low due to the lack of exploitation and recognition of the potential benefits of the new technology [7,8]. Consequently, entrepreneurs may not be maximizing the potential advantages IT could provide [9]. According to the Alternate Chair Digital Economy Working Group G20, in Indonesia, approximately 32 % of 64 million entrepreneurs are engaged online, indicating that 68 % of entrepreneurs have not adopted the digital realm for business development [10]. Entrepreneurs need to understand these benefits before implementing IT and realize that new technology is easy to use and useful for their business.

According to previous studies in the information systems field, social psychology theories, such as the theory of reasoned action (TRA) [11] and the theory of planned behavior [12], have impacted the study of individual attitudes as antecedents of user behavior [13,14]. Davis [15] built upon these theories to develop the technology acceptance model (TAM), elucidating behavior related to the use of technology. TAM posits that an individual's embrace of technology relies on two fundamental perceptions: perceived usefulness (PU) and perceived ease of use (PEOU) [15]. These perceptions are crucial in determining one's behavioral intentions and subsequent behavior toward using technology [13].

TAM posits that external factors can impact internal beliefs, attitudes, and intentions toward IT adoption [16]. Consistent with this, previous studies affirm that several factors play a role in an individual's decision to adopt technology [17]. One such variable that may be relevant to entrepreneurs is creativity, which can facilitate the formulation or creation of new ideas that were not previously available [18]. Creative entrepreneurs are expected to generate new ideas to help realize the potential benefits of new technology [19,20]. However, prior studies do not provide a comprehensive understanding of this ability, which may explain why some entrepreneurs are reluctant to use technology [21]. To fill this gap, our study introduces creativity as a key factor, expanding the scope of TAM to better explain the adoption of IT. In doing so, we aim to enhance our comprehension of how the model explains IT adoption and contributes to the theoretical advancement of this field.

Previous studies have highlighted the importance of recognizing the potential benefits of new technologies to improve their adoption and use [7,8]. However, the influence of a contextual factor in the relationship between creativity and both the PU and PEOU of a new technology should be determined. Entrepreneurial orientation (EO) is an appropriate contextual factor in previous empirical studies [22,23]. EO reflects the tendency to be innovative, proactive, and take risks, which can create an environment that supports creativity and adopting new technologies. Previous studies have explored individual attributes such as age and gender [24], experience, and IT skills [25], in addition to factors such as sample size, economic level, innovation level, and culture [26]. These factors are relatively broad compared to EO, although EO may be somewhat related to these contextual factors. EO delves deeper into substantial individual traits, particularly relevant to entrepreneurs who are the primary users of e-tax services and e-marketplaces in our investigation. In contrast to extant studies, EO is a better-suited variable for our study as it is more extensive. By understanding the moderating effects, we expect to enrich our comprehension of the connection between these variables and provide a more comprehensive insight into these relations.

This paper follows the subsequent structure: Section 2 introduces the literature review and hypothesis development. Section 3 contains the research methodology, including the measurement and research analysis. Section 4 presents the findings. Finally, Section 5 reveals the discussion, implications, limitations, and future research directions.

## 2 Literature review

### 2.1 Electronic service applications

Our research examines how people adopt new technologies by investigating the adoption of electronic service applications, with the main focus being the e-tax service and e-marketplace. E-marketplaces are applications used in the private sector to obtain services offered by private developers. In contrast, e-tax services are applications used in the public sector to obtain services provided by the government.

#### 2.1.1 E-tax service

E-tax service is an electronic government service that aims to improve the efficiency and effectiveness of government services by enhancing linkage and accessibility [27,28]. E-tax service facilitates the assessment, collection, and payment of taxes without physical interaction between the tax authorities and taxpayers [5,29]. This enables taxpayers to review, file, and pay taxes online.

Since 2007, the Directorate General of Taxes (DGT) in Indonesia has provided an official e-tax service application called DJP Online (https://djponline.pajak.go.id), which currently offers 16 services, including e-registration, e-filing, e-billing, taxpayer status confirmation, certificate of business domicile, e-objection, and e-reporting investment [30]. Taxpayers can use DJP Online to obtain the required tax services, among other available options.

#### 2.1.2 E-marketplace

E-marketplaces have become prominent tools for businesses to reach new customers and streamline their supply chain processes. They are defined as digital platforms connecting buyers and sellers, enabling them to conduct transactions and communicate [31]. According to the Indonesian Internet Service Providers Association, the most widely used e-marketplaces for selling products in Indonesia include Shopee (39 %), Tokopedia (24 %), Lazada (14 %), OLX (5 %), and Bukalapak (4 %) [32].

Three common classifications of e-marketplaces exist: independent, consortia-based, and private e-marketplaces [33]. Independent e-marketplaces are established and operated by third-party corporations, providing a platform for various suppliers to sell their products and services to a wide range of customers. Multiple large industry players develop consortia-based e-marketplaces to leverage their buying powers, share IT infrastructure, and reduce costs. Private e-marketplaces, as the name suggests, are developed and operated by a single large company to facilitate its own buying and selling activities [33]. Entrepreneurs, because of their limited resources, typically rely on existing independent e-marketplaces provided by digital companies.

### 2.2 Technology acceptance model

TAM is introduced as an adaptation of the TRA [16]. TRA explains that individuals' engagement in a specified behavior is influenced by their intention to engage in that behavior, which is jointly shaped by their attitude and subjective norms [16]. TAM was developed to explain why individuals accept or refuse new technology [34]. Previous studies have consistently utilized TAM, among other models, to comprehend the factors influencing an individual's engagement with technology [35].

**Fig. 1:**
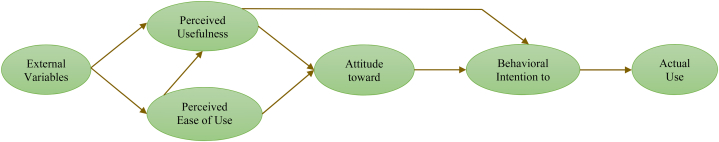
Technology acceptance model (TAM) (Davis, Bagozzi, Warshaw 1989).

As shown in Fig. 1, TAM proposes that individuals are inclined to either use or abstain from an application depending on their belief in its capacity to enhance their job performance. This belief is captured by the first TAM variable, PU. PU is described as the extent to which individuals believe that employing a specific technology would improve their job performance [36]. However, even if an application is useful, users may perceive the system as challenging to navigate, with the effort required to use the application outweighing the associated benefits. This belief is captured by PEOU, defined as the extent to which individuals believe that employing a specific technology would involve minimal effort [15,36]. TAM is a widely used model for explaining behavioral intention in information system research [16,21,33]. Thus, TAM will be used as the basis for the analytical framework in our study.

One of the key benefits of using TAM to understand technology usage behavior is that it includes other external variables that might affect PU and PEOU. External variables encompass system design features, user attributes (including cognitive style and other personality variables like creativity), task characteristics, nature of the development or implementation process, political influences, and organizational structures [16]. However, factors affecting new technology adoption may differ depending on the IT, intended users, and research context [37]. This insight is supported by research on applying TAM and external factors on technology acceptance [34].

#### 2.2.1 IT adoption and continuance intention

TAM proposes that the intention to adopt technology determines its actual utilization, which influences the technology continuance intention to use [16]. In our study, we investigate the intention to adopt IT among non-users with no prior experience of IT utilization and the continuance intention to use among the current users. Potential users develop their IT adoption intention based on the determinants of IT adoption—PU and PEOU—introduced by TAM [16]. Current users, however, develop IT continuance intention influenced by their initial acceptance decision, and their subsequent experiences with IT usage could potentially reverse their initial decisions [38]. Previous research has found that PU and PEOU are critical factors influencing IT adoption and continuance intention [34,39].

### 2.3 Creativity

Entrepreneurs must possess imagination and creativity to recognize the potential benefits of new technology for business processes. Shackle [40] emphasized the importance of imagination in uncertain situations, where entrepreneurs must evaluate different consequences, including implementing new technology. Business processes cannot be guided by foreknowledge; they are based solely on our anticipation of how they are likely to unfold [40].

Previous studies defined creativity as the ability to generate novel solutions and original ideas for problem-solving in any domain [18,19]. Stevenson et al. [41] and Zampetakis and Moustakis [42] similarly defined creativity as the ability to produce new and valuable ideas. Amabile et al. [43] highlighted the critical role of creativity in overcoming the obstacles that entrepreneurs encounter; thus, creativity is an essential skill that entrepreneurs must possess to improve their business performance [41,44,45].

### 2.4 Entrepreneurial orientation

According to Covin and Wales [46], EO is an organizational inclination toward entrepreneurial activities, including behavioral tendencies, managerial philosophies, and strategic decision-making practices [47]. EO encompasses managerial principles that aim to encourage innovative and learning-oriented behaviors, processes, methods, and styles [48]. Gupta and Gupta [49] also define EO as a set of entrepreneurial practices. While prior research has focused on EO at the organizational level, the original purpose behind developing EO was to distinguish individuals with entrepreneurial inclinations from those who are more conservative [23,50].

Miller [51] conceived EO as a concept that comprises three aspects, namely innovativeness, risk-taking, and proactiveness, which must exhibit a positive covariation for an EO to be evident [46]. Hence, EO should not be claimed when no covariation among these sub-dimensions exists according to the unidimensional view [52]. EO also encourages entrepreneurs to explore novel market opportunities and rejuvenate existing areas of operation [22]. Thus, it fosters a proactive approach to market opportunities, a willingness to tolerate risk, and an openness to innovation [22].

### 2.5 Hypotheses development

According to Davis et al. [16], entrepreneurs embrace the implementation of new technology when they perceive it as useful to improve their performance and easy to operate. Various studies have confirmed that PU and PEOU explain the behavioral intention and actual adoption behavior toward IT [3,16,34,53]. Therefore, non-user entrepreneurs who perceive new technology as valuable and user-friendly are more inclined to adopt it, while current users with elevated levels of PU and PEOU are more inclined to continue using IT in the future.

Furthermore, TAM suggests that an external variable (i.e., entrepreneurs’ creativity) directly influences PU and PEOU [16]. Creative entrepreneurs have the cognitive ability to generate novel ideas and perceive the use of technology as beneficial to their performance and not as difficult as they initially thought [19]. Therefore, creative entrepreneurs are expected to have a greater ability to recognize the usefulness and ease of use of IT applications than those who are not creative.

Based on the above information, we hypothesize that more creative entrepreneurs will have higher levels of PU and PEOU, leading to more IT adoption intention for non-users and IT continuance intention for current users. Specifically, we proposed the following hypotheses:

H1 PU positively mediates the relationship between creativity and IT adoption intention.

H2 PEOU positively mediates the relationship between creativity and IT adoption intention.

H3 PU positively mediates the relationship between creativity and IT continuance intention.

H4 PEOU positively mediates the relationship between creativity and IT continuance intention.

Previous research has indicated that entrepreneurs’ creativity positively impacts PU and ease of use in general [19]. However, for a stronger relationship between creativity and PU or PEOU, creative entrepreneurs must exhibit a greater willingness to take more calculated risks, demonstrate more innovation in their business, and be more proactive toward opportunities. These characteristics are associated with higher levels of EO, which may create an environment that is more receptive to adopting new technologies [23].

Moreover, research has found that individuals with higher levels of EO have different mental schemas than those without EO [23]. Entrepreneurially oriented individuals who are more open to novelty and change tend to be more receptive to the usefulness and ease of use of IT applications. Hence, the positive relationship between creativity and PU or PEOU will likely be amplified among entrepreneurs with a higher level of EO. Therefore, EO is suggested to boost the positive relationship between creativity and PU as well as creativity and PEOU [23]. Thus, we proposed the following hypotheses:

H5 EO will moderate the effect of creativity on PU, such that when EO is higher, the effect of creativity on PU is stronger.

H6 EO will moderate the effect of creativity on PEOU, such that when EO is higher, the effect of creativity on PEOU is stronger.

**Fig. 2:**
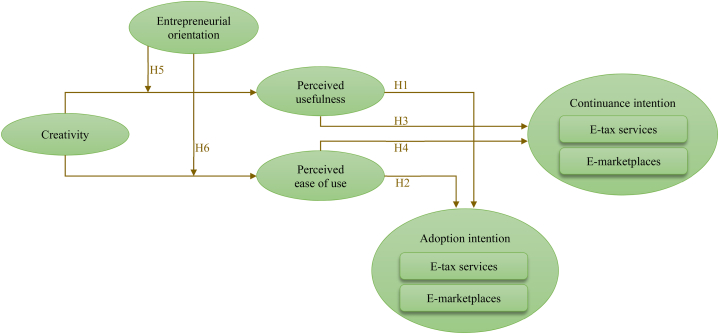
Conceptual framework.

Fig. 2 illustrates the conceptual framework employed in this study.

## 3 Methodology

### 3.1 Data collection

A survey questionnaire was used to collect data from entrepreneurs registered as individual taxpayers in the Yogyakarta Tax Office, Sleman Tax Office, Bantul Tax Office, Wates Tax Office, and Wonosari Tax Office, all of which are under the administration of the Regional Office of DGT for the Special Region of Yogyakarta, Indonesia. Initially, we created the survey in English and translated it into Indonesian using the back-translation method. We used the method because it is widely accepted for ensuring questionnaire equivalence between the source and our study. While there has been some debate about the effectiveness of back translation as a tool for testing translation quality in recent years, it still holds value as a documentation tool for explaining and justifying translation choices [54]. Consequently, we found this method suitable for our study. The sample of respondents were characterized as the top managers or owners of small and medium-sized enterprises with an annual gross revenue of less than or equal to 4.8 billion Indonesian rupiahs, or approximately 32,000 US dollars, as regulated by the government through DGT.

This region is unique as it maintains a blend of traditional economic activities, is well-suited to the countryside, and is a thriving educational hub. Yogyakarta is often called the “city of students,” as it hosts numerous higher education institutions, offering diverse avenues for enhancing economic activities by emphasizing digital applications.

This abundance of educational institutions also attracts students from various parts of Indonesia to study in Yogyakarta, fostering an environment where many entrepreneurs engage in digital business applications. Consequently, the entrepreneurial landscape in Yogyakarta strikes a balance between those who rely on traditional economic activities and those leveraging advanced IT systems in their business operations. Therefore, Yogyakarta serves as a representative and relevant microcosm of entrepreneurs in Indonesia.

The survey questionnaire was split into two sections: the first measured the adoption of the e-tax service, and the second measured the adoption of the e-marketplace. Different measurements were used for PU, PEOU, IT adoption intention, and IT continuance intention, with creativity and EO being used to measure both parts. The respondents were requested to complete both parts of the questionnaire to identify the different outcomes of e-tax service and e-marketplace adoption.

Before distributing the survey, permission was obtained from the DGT, and the heads of the Section of Internal Affairs of each designated tax office were contacted for data collection. Initially, we employed an online questionnaire to distribute the survey. However, owing to insufficient responses, we adapted our approach by disseminating surveys to entrepreneurs who visited designated tax offices. The questionnaire was then sent to the person-in-charge at the office to distribute to these taxpayers. After the person-in-charge collected the filled-in surveys, they were scanned and sent to researchers before the coding activities for the data analysis were conducted. Throughout this process, we faced constraints owing to the ongoing COVID-19 pandemic, which limited public mobility. Nevertheless, we successfully obtained the required number of responses. Therefore, it is challenging to repeat this data collection process.

### 3.2 Measurement

To measure creativity, we employed scales developed by Hills et al. [55] (for two items) and Puhakka [56] (the remaining items) that had previously been used to measure entrepreneurial creativity in other studies [57]. One of the items was, “I am a very creative person.” We employed creativity as the treatment variable for the matching analysis, creating a binary variable that indicated a high level of creativity with 1 and a low level of creativity with 0.

To measure the PU, PEOU, and IT adoption intention variables, we utilized four items for each variable and three items for IT continuance intention, all scales were developed by Davis et al. [16]. Sample items to measure the adoption of DJP Online included “I would find DJP Online useful for my business” for PU, “My interaction with DJP Online would be clear and understandable” for PEOU, “I intend to use DJP Online in the near future” for IT adoption intention, and “My intentions are to continue using DJP Online rather than use any alternative means (in-person services, by letter, etc.)” for IT continuance intention. Similarly, we also used these sample items: “Using e-marketplace enables me to accomplish tasks more quickly” for PU, “I would find e-marketplace is easy to operate” for PEOU, “I intend to continue using e-marketplace rather than discontinue its use” for IT continuance intention, and “I plan to use e-marketplace in the near future” for IT adoption intention to measure e-marketplace adoption. For measuring EO, we adapted eight items from Miller's [51] scale, including “My firm characteristically exhibits high levels of risk-taking, innovativeness, and proactiveness.”

All measures were self-reported and administered through a Likert-type scale, which comprised seven points from 1 (strongly disagree) to 7 (strongly agree). We also employed several demographic variables as covariates, including age, gender, educational background, marital status, type of industry, annual revenue, and number of employees.

### 3.3 Data analysis

Our research proposed a conceptual framework to analyze the relationship of creativity with IT adoption and continuance intention. We conducted two separate analyses to test this framework. In the initial phase, we explored the mediating effect of PU and PEOU on the relationship of creativity with IT adoption and continuance intention by conducting a causal mediation analysis. In the second stage, we investigated the moderating impact of EO on the relationship between creativity and PU, as well as between creativity and PEOU for IT adoption and continuance intention, by conducting a regression analysis.

To ensure the validity of our research method, we employed a technique called coarsened exact matching (CEM) based on previous studies. This technique helps reduce selection bias by ensuring a more balanced distribution of covariates between the treatment and control groups [[58], [59], [60], [61]]. King and Nielson [60] have indicated that another major matching method, propensity score matching (PSM), can lead to increased imbalance, inefficiency, reliance on the model, discretion in research, and statistical bias in both actual data and data specifically tailored to conform to the PSM theory. They also mentioned that as data balance improves, either naturally or through the removal of certain observations during the matching process, PSM tends to result in degraded inferences—a phenomenon known as the PSM paradox [60].

When the initial data exhibit significant imbalance and valid causal inferences are derived without heavy reliance on modeling assumptions, the paradox becomes preventable. However, this comes at the cost of rendering the data less valuable for causal inference through any method. Consequently, to address this challenge, CEM is introduced as an alternative approach. It helps us reduce selection bias by ensuring that the comparison groups are similar regarding certain observable characteristics. This improves our ability to determine the cause-and-effect relationships, particularly in situations where we cannot use controlled experiments [60].

We used the *cem* package to perform CEM in an R environment, following Iacus et al. [62]. According to the authors, CEM ensures that the imbalance between the treated and control groups stays within a chosen maximum limit. However, sometimes, the actual imbalance achieved through CEM may be less than the selected maximum. To comprehensively measure this imbalance, a multivariate imbalance metric is introduced, which is based on the absolute difference between multidimensional histograms of pre-treatment covariates in the treated and control groups. This method involves determining bin sizes for continuous variables, cross-tabulating discretized variables for both groups and then calculating the absolute difference between the relative frequencies in the matched sample. This measure provides a clearer evaluation of balance in matched datasets, aiding researchers in assessing the effectiveness of their matching procedures [62].

We estimated the causal effect of creativity on IT adoption and continuance intention and the mediation of PU and PEOU by incorporating matched weights of creativity, equalizing high-level and low-level creativity within each stratum [62]. Mediation analysis estimates the mechanisms that establish connections between independent and dependent variables [63]. We used the mediation package to compute the average mediation and direct effects by simulating predicted values for both the mediator and outcome variables (i.e., IT adoption and continuance intention). Thus, PU and PEOU mediate the relationships between creativity and IT adoption or continuance intention. Subsequently, we calculated the relevant quantities of interest, including average causal mediation effects (ACME), direct effects, and total effects [63].

To test the robustness of our results and investigate the violation of the sequential ignorability assumption, we conducted a sensitivity analysis [63]. To determine whether the true ACME significantly differs from zero, we initially assumed the presence of an unobserved confounding variable. This variable impacts both the mediators and outcomes consistently, causing the correlation between the two error terms to surpass or fall below the intersection point of the ACME at point 0 and the shaded area, denoting the confidence interval.

Finally, we conducted a moderation analysis to estimate the impact of EO on the association between creativity and PU as well as between creativity and PEOU for both e-tax service and e-marketplace adoption and continuance intention. EO acts as a moderator variable, influencing the direction and/or intensity of the relationship between creativity, which serves as an independent or predictor variable, and PU/PEOU, functioning as the dependent or criterion variable [24]. Before the moderation analysis, we computed the variance inflation factor (VIF) for the independent variables to detect multicollinearity and measure the extent of increase in the variance of the estimated coefficient of the variable due to multicollinearity with other independent variables in the model.

## 4 Result

### 4.1 Descriptive statistics

We administered the questionnaire to taxpayers who visited each of the chosen tax offices during a one-month period. Owing to the ongoing COVID-19 pandemic restrictions, there was a limited number of taxpayers present. Only those who met the predefined characteristics outlined in the data collection section were approached for participation. Tax office personnel supervised the survey and ensured the collection of completed surveys. Consequently, we obtained responses from all 272 questionnaires distributed, with 265 (97.43 %) of these responses deemed valid. Owing to these circumstances, we did not employ random sampling in this process. Invalid responses were due to incomplete or blank responses. Among the valid respondents, 159 (60 %) were men, and 106 (40 %) were women. Table 1A, 1B, 1C, and 1D display the mean, standard deviation, and correlations of all variables investigated in our study concerning the relationship between creativity and e-tax service adoption intention, creativity and e-tax service continuance intention, creativity and e-marketplace adoption intention, as well as creativity and e-marketplace continuance intention respectively.

**Table 1:** Means, standard deviations, and variables correlations for the main study variables.

A. E-tax service adoption intention														
Variables	M	SD	1	2	3	4	5	6	7	8	9	10	11	12
1. Age	35.12	8.65												
2. Gender	1.42	0.49	−0.11											
3. Education	3.27	0.86	0.08	0.30										
4. Marital status	1.33	0.47	−0.58	−0.05	−0.10									
5. Industry	1.35	0.48	0.15	−0.03	0.19	0.21								
6. Income	1.84	2.04	0.06	0.01	0.06	0.02	−0.05							
7. Size	1.43	0.99	0.16	−0.19	0.11	0.10	0.14	0.26						
8. Creativity	4.98	1.04	−0.08	−0.05	−0.03	0.03	0.15	0.09	0.16					
9. EO	4.42	0.85	−0.16	−0.10	−0.06	0.11	0.08	0.02	0.12	0.79				
10. PU	4.60	1.04	−0.20	−0.01	−0.02	0.13	0.08	0.03	0.01	0.68	0.71			
11. PEOU	4.69	1.06	−0.21	0.04	0.02	0.13	0.03	0.03	0.01	0.64	0.68	0.95		
12. Intention	4.33	1.07	−0.17	0.02	−0.07	0.10	0.17	0.03	0.10	0.52	0.52	0.57	0.58	

B. E-tax service continuance intention														
Variables	M	SD	1	2	3	4	5	6	7	8	9	10	11	12
1. Age	35.20	8.81												
2. Gender	1.38	0.49	−0.04											
3. Education	3.61	0.61	0.03	0.09										
4. Marital status	1.25	0.44	−0.36	0.16	−0.04									
5. Industry	1.37	0.48	0.06	0.20	0.02	0.07								
6. Income	1.48	1.33	0.12	−0.03	0.03	−0.05	0.14							
7. Size	1.73	0.92	0.03	0.05	0.10	0.08	0.13	0.35						
8. Creativity	5.14	1.20	−0.17	−0.16	0.01	−0.04	0.15	0.01	0.15					
9. EO	5.17	1.11	−0.18	−0.17	0.01	0.02	0.06	−0.04	0.10	0.84				
10. PU	5.30	1.11	−0.13	−0.05	−0.06	0.06	0.27	0.03	0.09	0.77	0.77			
11. PEOU	5.23	1.08	−0.18	0.01	−0.04	0.05	0.22	−0.02	0.07	0.77	0.79	0.95		
12. Intention	5.45	1.17	−0.11	−0.08	−0.02	0.03	0.23	0.03	0.05	0.66	0.71	0.84	0.87	

C. E-marketplace adoption intention														
Variables	M	SD	1	2	3	4	5	6	7	8	9	10	11	12
1. Age	35.80	8.80												
2. Gender	1.42	0.49	−0.12											
3. Education	3.35	0.83	0.04	0.19										
4. Marital status	1.30	0.46	−0.54	−0.02	−0.15									
5. Industry	1.38	0.49	0.13	0.01	0.12	0.17								
6. Income	1.85	2.04	0.03	−0.01	0.03	0.02	−0.02							
7. Size	1.52	1.05	0.14	−0.17	0.09	0.03	0.17	0.35						
8. CA	4.99	1.08	−0.03	−0.17	−0.06	0.02	0.21	0.10	0.20					
9. EO	4.79	0.96	−0.10	−0.19	−0.06	0.08	0.15	0.03	0.12	0.83				
10. PU	4.91	1.06	−0.15	−0.07	−0.02	0.12	0.14	0.05	0.05	0.76	0.83			
11. PEOU	4.42	1.02	−0.17	−0.05	−0.01	0.11	0.10	0.03	0.06	0.73	0.80	0.97		
12. Intention	4.54	1.08	−0.03	−0.04	−0.03	0.01	0.21	−0.01	0.15	0.66	0.66	0.74	0.72	

D. E-marketplace continuance intention														
Variables	M	SD	1	2	3	4	5	6	7	8	9	10	11	12
1. Age	34.16	8.53												
2. Gender	1.37	0.48	−0.02											
3. Education	3.60	0.60	0.16	0.24										
4. Marital status	1.27	0.45	−0.37	0.17	0.04									
5. Industry	1.33	0.47	0.05	0.23	0.16	0.08								
6. Income	1.34	0.96	0.22	0.01	0.14	−0.08	0.16							
7. Size	1.70	0.81	0.02	0.13	0.21	0.17	0.08	0.04						
8. Creativity	5.36	1.17	−0.25	−0.02	0.03	−0.06	0.07	−0.09	0.04					
9. EO	5.18	1.03	−0.26	−0.06	−0.01	0.02	−0.04	−0.07	0.08	0.84				
10. PU	5.53	0.97	−0.39	0.05	0.01	0.01	0.09	−0.18	−0.01	0.79	0.75			
11. PEOU	5.23	0.86	−0.41	0.04	0.01	−0.01	0.11	−0.19	−0.05	0.79	0.74	0.96		
12. Intention	5.23	0.95	−0.36	0.05	−0.04	0.01	0.04	−0.15	−0.03	0.73	0.73	0.85	0.89	

**Notes:** M = means, SD = standard deviation.

1 = Age, 2 = Gender (male and female), 3 = Education level (SD – elementary, SMP – junior high, SMA – senior high, D3/S1 – diploma/bachelor, S2/S3 – graduate), 4 = Marital status (married and not married), 5 = Industry type (trade and non-trade), 6 = Annual income (<250,000,000; 250,000,000 ≤ X < 500,000,000; 500,000,000 ≤ X < 750,000,000,; 750,000,000 ≤ X < 1,000,000,000; 1,000,000,000 ≤ X ≤ 1,250,000,000; 1,250,000,000 ≤ X ≤ 1,500,000,000; 1,500,000,000 ≤ X < 1,750,000,000; 1,750,000,000 ≤ X < 2,000,000,000; and ≥2,000,000,000), 7 = Business size (self-employed, 1–5, 6–10, 11–15, 16–20, >20), 8 = Creativity, 9 = Entrepreneurial orientation, 10 = Perceived usefulness, 11 = Perceived ease of use, 12 = Adoption/Continuance intention.

We performed assessments of convergent and discriminant validities for the main variables. We performed confirmatory factor analysis utilizing IBM SPSS AMOS version 29 to evaluate the measurement models. In this process, we scrutinized the factor loadings for each item. To gauge the overall goodness of fit of the model, we employed model-fit measures, all of which met commonly accepted standards [[64], [65], [66]], as illustrated in Appendix A, Table A1. To assess the reliability of the constructs, we utilized both Cronbach's alpha and composite reliability. The Cronbach's alpha values for all primary constructs exceeded 0.70, confirming the reliability of each construct, as detailed in Appendix A, Table A2 [67,68]. The convergent validity of the scale items was examined using the average variance extracted [69]. The average variance extracted values for all primary constructs exceeded the 0.50 threshold, as summarized in the same table, affirming the requisite convergent validity of the scales employed in this study. For assessing discriminant validity, we employed the Fornell and Lacker criterion and the heterotrait-monotrait ratio [69,70]. Most of the validity criteria were satisfied based on the Fornell and Lacker criterion. Moreover, when assessing the heterotrait-monotrait ratio (as shown in Appendix A Table A3 [70]), the ratios fell below the recommended threshold of 0.85 for all constructs but one pair, indicating good discriminant validity. The only exception was the ratio for PU and PEOU.

**Table 2:** Imbalance level between the treated and control groups before and after matching process.

Outcomes	**Multivariate distance (*L***_***1***_**)** before matching	**Multivariate distance (*L***_***1***_**)** after matching	*N* before matching	*N* after matching
e-tax service adoption intention	0.516	0.026	127	80
e-tax service continuance intention	0.530	0.000	138	86
e-marketplace adoption intention	0.494	0.000	162	112
e-marketplace continuance intention	0.580	0.286	103	63

**Table 3:** Mediation and sensitivity analysis results.

Variables	ACME	ADE	Total effect	**Value of *ρ* at which ACME 0**	**R**^**2**^**mediators**	**R**^**2**^**outcomes**	N
E-tax service adoption intention							
Creativity → PU → AD-ETS	0.501*** (0.192, 0.790)	0.283 (−0.156, 0.750)	0.784*** (0.378, 1.180)	0.4	0.398	0.330	80
Creativity → PEOU → AD-ETS	0.480*** (0.177, 0.820)	0.304 (−0.118, 0.680)	0.784*** (0.392, 1.150)	0.4	0.385	0.329	80
E-marketplace adoption intention							
Creativity → PU → AD-EMP	1.004*** (0.559, 1.510)	0.133 (−0.270, 0.460)	1.137*** (0.739, 1.270)	0.6	0.399	0.589	112
Creativity → PEOU → AD-EMP	0.813*** (0.418, 1.320)	0.324 (−0.118, 0.710)	1.137*** (0.735, 1.570)	0.6	0.339	0.544	112
E-tax service continuance intention							
Creativity → PU → CON-ETS	1.491*** (1.049, 1.990)	−0.063 (−0.401, 0.320)	1.428*** (1.005, 2.000)	0.8	0.566	0.801	86
Creativity → PEOU → CON-ETS	1.374*** (0.948, 1.900)	0.055 (−0.189, 0.300)	1.428*** (0.957, 1.160)	0.85	0.497	0.851	86
E-marketplace continuance intention							
Creativity → PU → CON-EMP	0.661*** (0.339, 1.050)	0.390* (0.045, 0.750)	1.051*** (0.751, 1.420)	0.7	0.598	0.813	63
Creativity → PEOU → CON-EMP	0.828*** (0.502, 1.220)	0.223 (−0.060, 0.520)	1.051*** (0.724, 1.410)	0.8	0.630	0.874	63

**Notes:** PU = perceived usefulness, PEOU = perceived ease of use, CON = continuance intention, AD = adoption intention, ETS = e-tax service, EMP = e-marketplace, ACME = average causal mediation effect, and ADE = average direct effect (95 % confident interval in parentheses).

Significant level: ***p < 0.001, **p < 0.01, *p < 0.05.

Our study used the congeneric approach to estimate the latent constructs [71,72]. This approach offers a more precise and dependable method by assigning distinct weights to each item, considering their variances, to estimate the latent variable [71].

### 4.2 CEM

We used gender, education level, marital status, industrial type, annual income, and firm size as covariates for the matching analysis. The global imbalance before and after the matching process is depicted in Table 2. The global imbalance measure (*L*_*1*_) before the matching as the baseline reference for the unmatched data compared with that after the matching process is reduced, suggesting an ideal equilibrium between the treated and untreated units regarding matched covariates. It shows a substantial reduction in imbalance due to the matching process, indicating that the imbalance in matching covariates before matching reduces for both the high- and low-level creativity groups.

### 4.3 Causal mediation analysis

Our research employs causal mediation analysis, followed by sensitivity analysis, for e-tax service and e-marketplace adoption intention and continuance intention, as detailed in Table 3 and depicted in Appendix C.

#### 4.3.1 Creativity, EO, PU/PEOU, and e-tax services adoption intention

The findings indicate a significant mediating impact of both PU and PEOU in the relationship between creativity and adoption intention, supported by ACME values of 0.501 (*p* < 0.001) and 0.480 (*p* < 0.001) for PU and PEOU, respectively. Conversely, the average direct effects (ADE) for both mediations are insignificant, signifying full mediation in the relationship. Moreover, the sensitivity analysis underscores the susceptibility of the ACME to variations in the creativity-adoption intention relationship. The results reveal that the *ρ* values for both PU and PEOU are 0.4 each, reinforcing the robustness of these findings [73]. Thus, these findings support H1 and H3.

#### 4.3.2 Creativity, EO, PU/PEOU, and e-tax services continuance intention

In this specific relationship, the findings also affirm full mediation, bolstered by significant ACME values of 1.491 (*p* < 0.001) for PU and 1.374 (*p* < 0.001) for PEOU, alongside the lack of significance in ADE within the relationship. Furthermore, the *ρ* values of 0.8 for PU and 0.85 for PEOU serve to underscore the relationship's robustness, as revealed through sensitivity analysis. Therefore, these findings support H2 and H4.

#### 4.3.3 Creativity, EO, PU/PEOU, and e-marketplace adoption intention

In this relationship, the results also indicate full mediation, supported by significant ACME values of 1.004 (*p* < 0.001) for PUand 0.813 (*p* < 0.001) for PEOU, as well as the insignificance of ADE in the relationship. The *ρ* values for both PU and PEOU, each register at 0.6, further reinforcing the robustness of the relationship through sensitivity analysis. Hence, these results also confirm H1 and H3.

#### 4.3.4 Creativity, EO, PU/PEOU, and e-marketplace continuance intention

Similar findings were observed for the relationship mediated by PEOU, indicated by an ACME value of 0.828 (*p* < 0.001) and no significance in the ADE within that relationship. However, in the case of the relationship mediated by PU, the ACME value is 0.661 (*p* < 0.001), while the ADE is 0.390 (p < 0.05), signifying partial mediation. The sensitivity analysis reaffirms the robustness of these relationships, with *ρ* values of 0.7 for the PU-mediated relationship and 0.8 for the PEOU-mediated relationship. Therefore, these findings also validate H2 and H4.

### 4.4 Moderation analysis

To analyze the hypotheses, we utilized the moderated hierarchical regression procedure suggested by Aiken and West [74] and Cohen et al. [75]. We included entrepreneurs’ age, gender, education level, marital status, industry type, annual income, and business size in the first step, creativity in the second step, EO in the third step, and interaction of creativity and EO in the fourth step. We first calculated the VIF for the independent variables, with the results revealing that none of the variables have a VIF greater than 5, indicating no substantial multicollinearity among the independent variables. All values of the mean VIF also support the conclusion that there is no significant issue of multicollinearity in the analysis.

We performed four multiple regression models for PU and PEOU for e-tax service, e-marketplace adoption, and continuance intention. Regression results for these variables are presented in Appendix D. Analyzing both types of applications and their adoption and continuance intentions provides additional insight. Generally, the interaction variable (creativity × EO) in the model shows that EO had no moderation effect on all relationships, except for that between creativity and PU for e-tax service adoption intention (β = 0.57, *p* < 0.05). The interaction terms shown in Table 4 support the significant result graphically. The figure presents the positive relationship between creativity and PU for entrepreneurs with a higher level of EO and the negative relationship for those with a lower level of EO. Therefore, H5 is supported only in the case of e-tax service adoption intentions. The other interaction terms in Appendix D indicate insignificant moderations in the other relationships. Thus, H6 was not supporte

**Table 4:** Moderation analysis.

	Moderation effect	N
E-tax services adoption intention		
Mediation of PU	0.57*	80
Mediation of PEOU	0.41	80
E-tax services continuance intention		
Mediation of PU	−0.34	86
Mediation of PEOU	−0.34	86
E-tax services adoption intention		
Mediation of PU	−0.10	112
Mediation of PEOU	−0.20	112
E-tax services continuance intention		
Mediation of PU	−0.27	63
Mediation of PEOU	−0.16	63

**Notes:** PU = Perceived usefulness; PEOU = Perceived ease of use.

Significant level: ***p < 0.001, **p < 0.01, *p < 0.05.

## 5 Discussion

The study's results confirm H1, H2, H3, and H4 regarding the mediation analyses. However, only H5 is supported for moderation effects, while H6 is not supported. Concerning the mediation analysis of PU and PEOU, the study found full and partial mediation effects.

The first finding reveals that PU and PEOU fully mediate the relationships between creativity and both the e-tax service and e-marketplace adoption intention and between creativity and e-tax service continuance intention. This indicates that creative entrepreneurs are more inclined to use the e-tax service and e-marketplace if they perceive them to be fun and beneficial for their productivity. The results confirm previous findings on the indirect impact of PU and PEOU on the relationship between creativity and entrepreneurial intention behaviors for exploiting innovative learning tools [19].

The second finding reveals the partial mediation effects of PU on the relationship between creativity and e-tax service continuance intention. This indicates that PU directly and indirectly impacts the relationship. The mediation effect of PU explains 58 % of the variation. This implies that although creative entrepreneurs are more inclined to perceive that the e-tax service is easy to use and useful to them and that the e-marketplace helps enhance their performance, they will be more likely to continue using the e-marketplace—and it may be necessary to explain the variation in terms of the existence of other potential mediating factors. Prior research suggests that entrepreneurial self-efficacy, attitude, subjective norms [76], and learning [19] partially mediate the relationship between entrepreneurs’ creativity and entrepreneurial behavioral outcomes. However, further investigation is necessary to discern whether PU, PEOU, and other mediators operate in parallel rather than subsequent processes.

Finally, the study found a moderating impact of EO to strengthen the relationship between creativity and PU for e-tax service adoption intention. This indicates that EO had a notable influence on changing the direction of the relationship between entrepreneurs’ creativity and PU for non-e-tax service users who are willing to adopt the application in the future. This confirms the findings of previous studies, which revealed that EO offers a particular context to boost the relationship between creativity and PU [22].

However, the study failed to find support for the moderating impact of EO on other relationships. EO does not have a significant effect on changing the direction or strength of the relationships between entrepreneurs' creativity and PU, as well as PEOU for e-tax service and e-marketplace adoption and continuance intention, except for PU for e-tax service adoption intention, as mentioned above. The findings reveal that entrepreneurs’ creativity positively influences PU and PEOU for e-tax service continuance intention as well as for e-marketplace adoption and continuance intention regardless of EO levels.

## 6 Theoretical implications

This research significantly contributes to the field of creativity and technology adoption by offering several theoretical implications. First, unlike prior studies that predominantly focused on technology's PU and PEOU, our study brings attention to the role of entrepreneurs' creativity as a crucial external variable. By extending the TAM to include creativity as an antecedent variable, we shed light on a previously unexplored dimension. It is essential to acknowledge that the TAM framework, lacking external variables, simplifies the view of technology acceptance by concentrating on individual perceptions and overlooking broader contextual factors, especially user characteristics influencing adoption. However, previous studies have failed to investigate the factors impacting users' acceptance of new technology, particularly regarding how they perceive its usefulness and ease of use when TAM does not consider related external variables. Furthermore, Davis et al. [16] suggested the applicability of several external variables in the TAM framework. The study's findings contribute to the existing TAM by extending it to include creativity as an antecedent variable, making entrepreneurs' creativity a worthwhile area for analysis due to its impact on adopting new IT.

Examining the exogenous variables, such as creativity, that affect the fundamental constructs of TAM helps provide a better understanding of intention and actual usage behaviors [21], as uniquely demonstrated in our study. In contrast with factors identified in extant studies, we explain that the variance in PU is attributed to creativity to a greater extent. This finding becomes evident when considering factors that contribute to variance to a lesser degree, such as trust [77], optimism, innovativeness, insecurity, and discomfort [77,78]. This is particularly notable in the context of the relationship between creativity and the intentions to both adopt and continue using e-tax services and e-marketplaces. Similarly, we found that creativity explains a larger portion of the variance in PEOU than that found in extant studies. This is evident when we compare the explained variances in PEOU in our study with those in previous research, such as those in self-awareness [79], trust [77], optimism, innovativeness, insecurity, and discomfort [78]. This effect is particularly pronounced in the context of the relationship between creativity and the intention to adopt e-tax services and the intention to continue using e-tax services and e-marketplaces. This means that we are broadening the scope of the model by considering how external factors such as creativity can influence the core elements of TAM, which include PU, PEOU, and individuals’ intentions and actual behaviors in adopting and using technology [19]. Our findings provide a more comprehensive understanding of how creativity plays a role in shaping technology adoption.

Thus, creativity plays a crucial role in the acceptance of technology, especially when compared to other factors previously mentioned. It is instrumental in helping entrepreneurs recognize technology's potential benefits by fostering new ideas and innovative solutions [42,45]. Creative individuals can think of unique paths and possibilities, adding diverse perspectives to how technology is adopted. Additionally, creative individuals show adaptability and flexibility in their thinking, which is crucial when dealing with new technologies that may require adjustments to established habits or workflows [42]. The ability to navigate and welcome change is a key aspect of creativity, providing users with valuable skills to effectively incorporate new technological advancements into their daily routines. Creativity is closely connected to effective problem-solving skills [80]. In the context of technology, users who approach challenges with a creative mindset may be better equipped to overcome issues related to technology use. The ever-changing nature of technology often brings complex problems, and creative individuals who can think beyond conventional boundaries can contribute fresh and efficient problem-solving approaches, making the adoption process smoother [42].

Second, the study examines how creativity affects two types of behavioral intentions (adoption and continuance) in two different contexts (e-tax service and e-marketplace) through the mediating factors of PU and PEOU. The TAM framework is expanded to include creativity and explain how it influences entrepreneurs' behavior. The study revealed that the effects of creativity on adoption intention are stronger than those on continuance intention through both PU and PEOU in the e-marketplace context. This suggests that entrepreneurs need to generate more innovative ideas concerning the benefits and ease of using a new e-marketplace, especially when they lack prior experience with it. As entrepreneurs become familiar with the technology, the need for creativity diminishes because they have already recognized the e-marketplace's advantages. Additionally, the effects of creativity on the e-marketplace are stronger than those on the e-tax service through both PU and PEOU in the adoption intention context. This suggests that entrepreneurs perceive their creativity as more impactful in enhancing the usefulness and ease of use of an e-marketplace than an e-tax service. Entrepreneurs may perceive e-marketplaces as more beneficial to enhancing their business profitability while viewing e-tax services as primarily impacting cost efficiency. The study fills a gap in TAM research by empirically demonstrating the mediation effects of PU and PEOU in the relationship of creativity with IT adoption and continuance. Previous literature has mainly focused on adoption intention [19], which limits the comparison of the effects of creativity between potential and current users. By considering the mediating roles of PU and PEOU, we move beyond intention and instead explore the actual experiences of users. This shift in focus allows us to assess how creativity affects both potential and current users, providing a more comprehensive perspective.

Finally, the research investigated how EO influences the relationships between creativity and PU/PEOU in terms of adopting and continuing to use the e-tax service and e-marketplace scenarios. The results revealed that EO strengthens the relationship between creativity and PU regarding an intention to adopt an e-tax service. This addresses a gap in TAM research by highlighting the moderating effect of EO. This effect is demonstrated in the relationship between creativity and PU, which has received scarce attention in previous research. We explore how an individual's entrepreneurial mindset or disposition can influence how creativity impacts their perception of a technology's usefulness. This unique contribution adds depth to our understanding of the elements affecting the adoption and utilization of technology. Our study stands out in the field by emphasizing the examination of moderation effects, a facet often overlooked in previous research. Understanding moderation effects is of paramount significance as they unveil how the impact of one variable on another can vary under different circumstances. This deepens our comprehension and understanding of the relationships between the variables.

## 7 Practical implications

The results of our study offer a crucial managerial guideline for exploring the factors that influence technology adoption among entrepreneurs. It is crucial for managers who provide electronic services, such as e-tax services or e-marketplaces, to focus on encouraging creativity among entrepreneurs because it significantly contributes to the success of technology adoption efforts. Recognizing creativity as a critical external variable in TAM implies that strategies should be directed toward fostering a conducive environment for creative thinking among entrepreneurs. This not only influences how entrepreneurs perceive the user interface of applications but also empowers them to identify technical features that can elevate their business operations.

Addressing individuals' creative potential encompasses three key components of individual creativity, according to Amabile et al.’s [38] componential theory of individual creativity. The elements consist of proficiency, creative-thinking skill, and intrinsic task motivation [19]. These components are essential for fostering creativity in any specific domain. The theory posits that creativity will likely appear when individuals' skills align with their most profound intrinsic interests or strongest passions. Additionally, creativity increases as proficiency in each of these three components rises. Put differently, individuals with higher levels of skills, motivation, and creative thinking are more likely to exhibit creativity in their specific domain of interest.

Based on this theory, managers providing electronic services should prioritize promoting individual creativity development. This can be achieved through various strategies outlined in prior research as practical approaches. For instance, managers could offer training and development programs such as workshops to enhance skills relevant to the e-marketplace or tax management. These programs should cover areas such as digital marketing, financial management, and technological advancements, empowering entrepreneurs particularly to think creatively about their business strategies. Certification programs validating expertise in utilizing e-marketplace platforms or navigating tax compliance can also be introduced, enhancing credibility, and encouraging a commitment to continuous learning and innovation. Additionally, mentorship programs involving experienced entrepreneurs or industry experts can offer personalized insights and stimulate creative problem-solving.

Another avenue for managers is to offer a platform for collaboration, where entrepreneurs can collaborate with other entrepreneurs and experts in their field [81]. These virtual spaces could include discussion forums, webinars, or collaborative projects, facilitating the sharing of insights and innovative ideas related to the digital marketplace or tax services. Interaction with experts in e-commerce, taxation, and related fields can provide valuable guidance for navigating the digital business landscape and staying compliant with tax regulations. More importantly, in our context, building a sense of community by encouraging entrepreneurs to share success stories, challenges, and solutions can inspire creativity as entrepreneurs learn from each other's experiences.

Furthermore, managers could provide entrepreneurs access to resources, such as industry reports, market research, and other relevant information within the e-marketplace or e-tax service platform. This information can empower entrepreneurs to make informed decisions and identify new opportunities for innovation. Access to legal and regulatory updates can also be provided to keep entrepreneurs informed about changes in tax regulations and e-commerce laws. Access to a library of best practices in e-commerce and tax management may inspire creativity by allowing entrepreneurs to draw inspiration from successful case studies and adopt proven strategies to optimize their business operations.

Finally, managers can enhance the culture of innovation among the entrepreneurs by recognizing and rewarding entrepreneurs who demonstrate creativity in their activities. Innovation awards within their e-services platform can acknowledge entrepreneurs who creatively utilize the marketplace or effectively manage their tax processes, motivating others to approach technology adoption with creativity. Virtual showcases or exhibitions can provide a platform for creative entrepreneurs to demonstrate their products, services, or innovative business models, fostering healthy competition and inspiration within the entrepreneurial community.

## 8 Limitations and future research

The research has some limitations that offer potential for future studies. First, because we relied on subjective measures, as reported by the entrepreneurs themselves, to gauge their creativity, EO, and other technology adoption outcomes, future studies should employ more objective measures that have been validated in previous research.

Second, our study was conducted solely on entrepreneurs who were registered as taxpayers in a small number of tax offices, resulting in a limited sample size. To increase the number of responses, future research should obtain data from alternative sources related to entrepreneurship, such as entrepreneurial cooperatives, organizations, and associations. This would result in a more diverse and representative sample, thereby enhancing the generalizability of the findings.

Third, because entrepreneurs’ demographic data were unavailable, we could not perform a balance check between the respondents and non-respondents in the population. In the future, we recommend obtaining this data to facilitate a balance check.

Finally, despite using CEM to mitigate selection bias, it is crucial to acknowledge that matching is not a flawless method for addressing selection bias as it might not account for unobserved covariates. We propose considering randomized controlled trials as a potential avenue for future research.

## Acknowledment

The funding for this research was provided by The Indonesia Endowment Fund for Education, grant number S-710/LPDP.4/2023.

## Data availability statement

Related data have been published online at https://doi.org/10.17632/kcvpw2pxh6.1.

## Ethical approval statement

The Ethics Committee of the Graduate School of Humanities and Social Sciences of Hiroshima University, Japan, reviewed and approved on February 3, 2022. All participants provided written informed consent, and the questionnaires were anonymized. Respondents had the option to withdraw from the survey at any point if they felt uncomfortable.

## CRediT authorship contribution statement

**Adin Kusumo Putro:** Writing – original draft, Visualization, Validation, Software, Resources, Project administration, Methodology, Investigation, Funding acquisition, Formal analysis, Data curation, Conceptualization. **Yoshi Takahashi:** Writing – review & editing, Validation, Supervision, Software, Resources, Methodology, Investigation, Conceptualization.

## Declaration of competing interest

The authors declare that they have no known competing financial interests or personal relationships that could have appeared to influence the work reported in this paper.
