# Supplementary material for: Entrepreneurs’ creativity, information technology adoption, and continuance intention: Mediation effects of perceived usefulness and ease of use and the moderation effect of entrepreneurial orientation
Source: Heliyon. 2024 Feb 2;10(3):e25479. doi: 10.1016/j.heliyon.2024.e25479 (PMC10865247; doi:10.1016/j.heliyon.2024.e25479)
Supplement: Multimedia component 1 [file mmc1.docx]

**Appendix A**

**Table A.1.** Confirmatory factor analysis

| Fit Indices | Recommended Value | A | B | C | D |
| --- | --- | --- | --- | --- | --- |
| CMIN/df | Less than 2 [63] to 5 [68] | 2.103 | 2.004 | 2.442 | 1.804 |
| GFI | >.90 [60] | 0.751 | 0.763 | 0.776 | 0.740 |
| CFI | >.90 [61] | 0.917 | 0.945 | 0.923 | 0.941 |
| TLI | >.90 [61] | 0.899 | 0.935 | 0.905 | 0.930 |
| SRMR | <.80 [62] | 0.051 | 0.031 | 0.044 | 0.037 |
| RMSEA | <.80 [62] | 0.094 | 0.086 | 0.095 | 0.089 |
| **Notes:** *A = Creativity, EO, PU, PEOU, and e-tax services adoption intention; B = Creativity, EO, PU, PEOU, and e-tax services continuance intention; C = Creativity, EO, PU, PEOU, and e-marketplace adoption intention; D = Creativity, EO, PU, PEOU, and e-marketplace continuance intention.* | | | | | |

**Table A.2** Reliability and convergent validity

| Items | Cronbach’s alpha | | | | Composite reliability | | | | AVE | | | |
| --- | --- | --- | --- | --- | --- | --- | --- | --- | --- | --- | --- | --- |
|  | A | B | C | D | A | B | C | D | A | B | C | D |
| *Creativity* | 0.937 | 0.969 | 0.944 | 0.972 | 0.939 | 0.969 | 0.943 | 0.973 | 0.658 | 0.798 | 0.676 | 0.817 |
| *Entrepreneurial orientation* | 0.939 | 0.960 | 0.938 | 0.966 | 0.944 | 0.959 | 0.939 | 0.967 | 0.677 | 0.745 | 0.659 | 0.788 |
| *Perceived usefulness* | 0.938 | 0.968 | 0.943 | 0.975 | 0.942 | 0.975 | 0.946 | 0.976 | 0.802 | 0.897 | 0.814 | 0.912 |
| *Perceived ease of use* | 0.955 | 0.966 | 0.954 | 0.967 | 0.957 | 0.965 | 0.959 | 0.964 | 0.848 | 0.875 | 0.853 | 0.870 |
| *Adoption/continuance intention* | 0.962 | 0.975 | 0.976 | 0.963 | 0.963 | 0.972 | 0.976 | 0.963 | 0.896 | 0.896 | 0.933 | 0.868 |
| **Notes:** *A = Creativity, EO, PU, PEOU, and E-tax services Adoption Intention; B = Creativity, EO, PU, PEOU, and E-tax services Continuance Intention; C = Creativity, EO, PU, PEOU, and E-marketplace Adoption Intention; D = Creativity, EO, PU, PEOU, and E-marketplace Continuance Intention.* | | | | | | | | | | | | |

**Table A.3.** Discriminant validity

| A. Creativity, EO, PU, PEOU, and e-tax services adoption intention | |
| --- | --- |
| Fornell and Larcker criterion   \|  \| **CA** \| **EO** \| **PU** \| **PEOU** \| **ADP** \| \| --- \| --- \| --- \| --- \| --- \| --- \| \| **CA** \| 0.811 \|  \|  \|  \|  \| \| **EO** \| 0.761 \| 0.823 \|  \|  \|  \| \| **PU** \| 0.658 \| 0.691 \| 0.896 \|  \|  \| \| **PEOU** \| 0.616 \| 0.658 \| ***0.922*** \| 0.921 \|  \| \| **ADP** \| 0.499 \| 0.502 \| 0.548 \| 0.564 \| 0.896 \| | Heterotrait-Monotrait (HTMT) Ratio   \|  \| **CA** \| **EO** \| **PU** \| **PEOU** \| **ADP** \| \| --- \| --- \| --- \| --- \| --- \| --- \| \| **CA** \|  \|  \|  \|  \|  \| \| **EO** \| 0.769 \|  \|  \|  \|  \| \| **PU** \| 0.658 \| 0.700 \|  \|  \|  \| \| **PEOU** \| 0.617 \| 0.667 \| ***0.925*** \|  \|  \| \| **ADP** \| 0.499 \| 0.508 \| 0.549 \| 0.566 \|  \| |

| B. Creativity, EO, PU, PEOU, and e-tax services continuance intention | |
| --- | --- |
| Fornell and Larcker criterion   \|  \| **CA** \| **EO** \| **PU** \| **PEOU** \| **CON** \| \| --- \| --- \| --- \| --- \| --- \| --- \| \| **CA** \| 0.893 \|  \|  \|  \|  \| \| **EO** \| 0.814 \| 0.863 \|  \|  \|  \| \| **PU** \| 0.759 \| 0.753 \| 0.947 \|  \|  \| \| **PEOU** \| 0.750 \| 0.771 \| 0.929 \| 0.935 \|  \| \| **CON** \| 0.638 \| 0.692 \| 0.817 \| 0.850 \| 0.947 \| | Heterotrait-Monotrait (HTMT) Ratio   \|  \| **CA** \| **EO** \| **PU** \| **PEOU** \| **CON** \| \| --- \| --- \| --- \| --- \| --- \| --- \| \| **CA** \|  \|  \|  \|  \|  \| \| **EO** \| 0.808 \|  \|  \|  \|  \| \| **PU** \| 0.764 \| 0.754 \|  \|  \|  \| \| **PEOU** \| 0.749 \| 0.766 \| ***0.936*** \|  \|  \| \| **CON** \| 0.634 \| 0.684 \| 0.818 \| 0.844 \|  \| |

| C. Creativity, EO, PU, PEOU, and e-marketplace adoption intention | |
| --- | --- |
| Fornell and Larcker criterion   \|  \| **CA** \| **EO** \| **PU** \| **PEOU** \| **ADP** \| \| --- \| --- \| --- \| --- \| --- \| --- \| \| **CA** \| 0.822 \|  \|  \|  \|  \| \| **EO** \| 0.792 \| 0.812 \|  \|  \|  \| \| **PU** \| 0.731 \| 0.802 \| 0.902 \|  \|  \| \| **PEOU** \| 0.707 \| 0.779 \| ***0.950*** \| 0.924 \|  \| \| **ADP** \| 0.642 \| 0.638 \| 0.725 \| 0.707 \| 0.966 \| | Heterotrait-Monotrait (HTMT) Ratio   \|  \| **CA** \| **EO** \| **PU** \| **PEOU** \| **ADP** \| \| --- \| --- \| --- \| --- \| --- \| --- \| \| **CA** \|  \|  \|  \|  \|  \| \| **EO** \| 0.788 \|  \|  \|  \|  \| \| **PU** \| 0.730 \| 0.805 \|  \|  \|  \| \| **PEOU** \| 0.708 \| 0.784 \| ***0.960*** \|  \|  \| \| **ADP** \| 0.638 \| 0.638 \| 0.728 \| 0.711 \|  \| |

| D. Creativity, EO, PU, PEOU, and e-marketplace continuance intention | |
| --- | --- |
| Fornell and Larcker criterion   \|  \| **CA** \| **EO** \| **PU** \| **PEOU** \| **CON** \| \| --- \| --- \| --- \| --- \| --- \| --- \| \| **CA** \| 0.904 \|  \|  \|  \|  \| \| **EO** \| 0.820 \| 0.888 \|  \|  \|  \| \| **PU** \| 0.777 \| 0.733 \| 0.955 \|  \|  \| \| **PEOU** \| 0.766 \| 0.720 \| ***0.938*** \| 0.933 \|  \| \| **CON** \| 0.709 \| 0.743 \| 0.833 \| 0.872 \| 0.932 \| | Heterotrait-Monotrait (HTMT) Ratio   \|  \| **CA** \| **EO** \| **PU** \| **PEOU** \| **CON** \| \| --- \| --- \| --- \| --- \| --- \| --- \| \| **CA** \|  \|  \|  \|  \|  \| \| **EO** \| 0.822 \|  \|  \|  \|  \| \| **PU** \| 0.779 \| 0.736 \|  \|  \|  \| \| **PEOU** \| 0.762 \| 0.717 \| ***0.935*** \|  \|  \| \| **CON** \| 0.709 \| 0.744 \| 0.835 \| 0.867 \|  \| |

**Notes:** *CA = Creativity; EO = Entrepreneurial orientation; PU = Perceived usefulness; PEOU = Perceived ease of use; ADP = Adoption intention; CON = Continuance intention*

**Appendix B.** Imbalance level

**Table B.1:** Creativity, EO, PU/PEOU and e-tax services adoption intention

|  | Before matching | | | | | | | After matching | | | | | | |
| --- | --- | --- | --- | --- | --- | --- | --- | --- | --- | --- | --- | --- | --- | --- |
|  | *L_1_* | *mean* | *Min* | *25%* | *50%* | *75%* | *Max* | *L_1_* | *mean* | *Min* | *25%* | *50%* | *75%* | *Max* |
| Multivariate L_1_ distance | 0.516 |  |  |  |  |  |  | 0.026 |  |  |  |  |  |  |
| Gender | 0.098 | 0.098 | 0 | 0 | 0 | 0 | 0 | 0.000 | 0.000 | 0 | 0 | 0 | 0 | 0 |
| Education | 0.061 | 0.105 | 0 | 0 | 0 | 0 | 0 | 0.000 | 0.000 | 0 | 0 | 0 | 0 | 0 |
| Marital status | 0.134 | -0.134 | 0 | 0 | 0 | 0 | 0 | 0.000 | 0.000 | 0 | 0 | 0 | 0 | 0 |
| Industry | 0.176 | -0.176 | 0 | 0 | 0 | 0 | 0 | 0.000 | 0.000 | 0 | 0 | 0 | 0 | 0 |
| Income | 0.089 | -0.332 | 0 | 0 | 0 | -1 | 0 | 0.068 | 0.026 | 0 | 0 | 0 | 0 | 1 |
| Size | 0.053 | -0.187 | 0 | 0 | 0 | -1 | -1 | 0.000 | 0.000 | 0 | 0 | 0 | 0 | 0 |
| *N* | 127 | | | | | | | 80 | | | | | | |

**Table B.2:** Creativity, EO, PU/PEOU and e-tax services continuance intention

|  | Before matching | | | | | | | After matching | | | | | | |
| --- | --- | --- | --- | --- | --- | --- | --- | --- | --- | --- | --- | --- | --- | --- |
|  | *L_1_* | *mean* | *Min* | *25%* | *50%* | *75%* | *Max* | *L_1_* | *mean* | *Min* | *25%* | *50%* | *75%* | *Max* |
| Multivariate L_1_ distance | 0.530 |  |  |  |  |  |  | 0.000 |  |  |  |  |  |  |
| Gender | 0.165 | -0.165 | 0 | 0 | 0 | 0 | 0 | 0.000 | 0.000 | 0 | 0 | 0 | 0 | 0 |
| Education | 0.135 | 0.003 | 2 | 0 | 0 | 0 | 0 | 0.000 | 0.000 | 0 | 0 | 0 | 0 | 0 |
| Marital status | 0.036 | 0.036 | 0 | 0 | 0 | 1 | 0 | 0.000 | 0.000 | 0 | 0 | 0 | 0 | 0 |
| Industry | 0.081 | 0.081 | 0 | 0 | 0 | 0 | 0 | 0.000 | 0.000 | 0 | 0 | 0 | 0 | 0 |
| Income | 0.041 | -0.157 | 0 | 0 | 0 | 0 | -1 | 0.000 | -0.109 | 0 | 0 | 0 | -1 | 0 |
| Size | 0.288 | 0.210 | 0 | 0 | 1 | 0 | 0 | 0.000 | 0.000 | 0 | 0 | 0 | 0 | 0 |
| *N* | 138 | | | | | | | 86 | | | | | | |

**Table B.3:** Creativity, EO, PU/PEOU and e- marketplace adoption intention

|  | Before matching | | | | | | | After matching | | | | | | |
| --- | --- | --- | --- | --- | --- | --- | --- | --- | --- | --- | --- | --- | --- | --- |
|  | *L_1_* | *mean* | *Min* | *25%* | *50%* | *75%* | *Max* | *L_1_* | *mean* | *Min* | *25%* | *50%* | *75%* | *Max* |
| Multivariate L_1_ distance | 0.494 |  |  |  |  |  |  | 0.000 |  |  |  |  |  |  |
| Gender | 0.119 | 0.119 | 0 | 0 | -1 | 0 | 0 | 0.000 | 0.000 | 0 | 0 | 0 | 0 | 0 |
| Education | 0.042 | 0.053 | 0 | 0 | 0 | 0 | 0 | 0.000 | 0.000 | 0 | 0 | 0 | 0 | 0 |
| Marital status | 0.069 | -0.069 | 0 | 0 | 0 | 1 | 0 | 0.000 | 0.000 | 0 | 0 | 0 | 0 | 0 |
| Industry | 0.114 | -0.114 | 0 | 0 | 0 | 0 | 0 | 0.000 | 0.000 | 0 | 0 | 0 | 0 | 0 |
| Income | 0.064 | -0.008 | 0 | 0 | 0 | 1 | 0 | 0.000 | -0.044 | 0 | 0 | 0 | 0 | 0 |
| Size | 0.069 | -0.158 | 0 | 0 | 0 | 1 | 1 | 0.000 | 0.000 | 0 | 0 | 0 | 0 | 0 |
| *N* | 162 | | | | | | | 112 | | | | | | |

**Table B.4:** Creativity, EO, PU/PEOU and e- marketplace continuance intention

|  | Before matching | | | | | | | After matching | | | | | | |
| --- | --- | --- | --- | --- | --- | --- | --- | --- | --- | --- | --- | --- | --- | --- |
|  | *L_1_* | *mean* | *Min* | *25%* | *50%* | *75%* | *Max* | *L_1_* | *mean* | *Min* | *25%* | *50%* | *75%* | *Max* |
| Multivariate L_1_ distance | 0.580 |  |  |  |  |  |  | 0.286 |  |  |  |  |  |  |
| Gender | 0.101 | -0.101 | 0 | 0 | 0 | 0 | 0 | 0.000 | 0.000 | 0 | 0 | 0 | 0 | 0 |
| Education | 0.060 | 0.052 | 2 | 0 | 0 | 0 | 0 | 0.000 | 0.000 | 0 | 0 | 0 | 0 | 0 |
| Marital status | 0.057 | 0.057 | 0 | 0 | 0 | 1 | 0 | 0.000 | 0.000 | 0 | 0 | 0 | 0 | 0 |
| Industry | 0.075 | 0.075 | 0 | 0 | 0 | 0 | 0 | 0.000 | 0.000 | 0 | 0 | 0 | 0 | 0 |
| Income | 0.048 | -0.230 | 0 | 0 | 0 | 0 | -6 | 0.057 | -0.057 | 0 | 0 | 0 | 0 | 0 |
| Size | 0.242 | 0.095 | 0 | 0 | 1 | 0 | -3 | 0.000 | 0.000 | 0 | 0 | 0 | 0 | 0 |
| *N* | 103 | | | | | | | 63 | | | | | | |

**Appendix C.** Sensitivity analysis of the mediation analysis

**Figure C.1:** Creativity, EO, PU/PEOU and e-tax services adoption intention

| Perceived usefulness | Perceived ease of use |
| --- | --- |
| 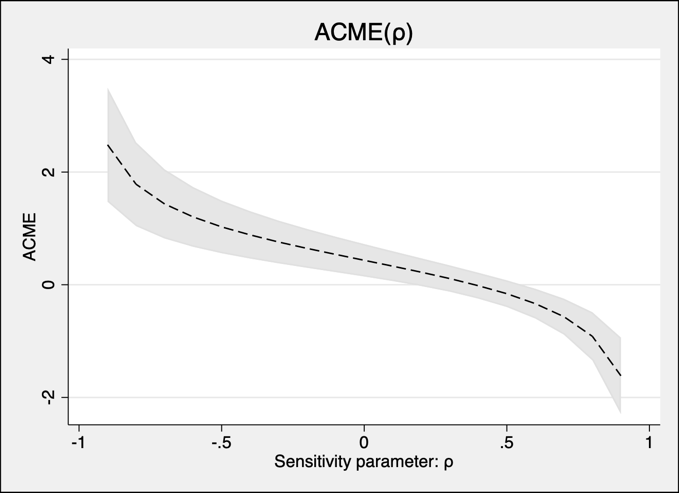 |  |

**Figure C.2:** Creativity, EO, PU/PEOU and e-tax services continuance intention

| Perceived usefulness | Perceived ease of use |
| --- | --- |
|  |  |

**Figure C.3:** Creativity, EO, PU/PEOU and e- marketplace adoption intention

| Perceived usefulness | Perceived ease of use |
| --- | --- |
| 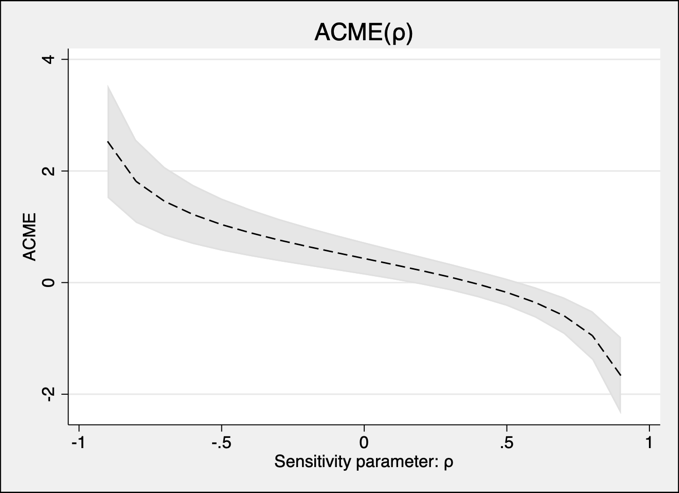 |  |

**Figure C.3:** Creativity, EO, PU/PEOU and e- marketplace continuance intention

| Perceived usefulness | Perceived ease of use |
| --- | --- |
|  |  |

**Notes:** ACME: average causal mediation effect

**Appendix D.** Moderation analysis

**Table D.1:**  Moderated hierarchical regressions toward e-tax service adoption intention

| Variables | Perceived usefulness | | | | Perceived ease of use | | | |
| --- | --- | --- | --- | --- | --- | --- | --- | --- |
|  | Model 1 | Model 2 | Model 3 | Model 4 | Model 5 | Model 6 | Model 7 | Model 8 |
| Creativity |  | 1.18*** | 0.13 | -2.43* |  | 1.16*** | 0.22 | -1.63 |
| Entrepreneurial orientation |  |  | 0.84*** | 0.63*** |  |  | 0.76*** | 0.60*** |
| CA X EO |  |  |  | 0.57* |  |  |  | 0.41 |
| Age | -0.01 | 0.01 | -0.01 | -0.01 | -0.01 | 0.01 | -0.01 | -0.01 |
| Gender | 0.02 | 0.06 | 0.01 | 0.07 | 0.15 | 0.19 | 0.14 | 0.19 |
| Education level | 0.12 | 0.12 | 0.04 | -0.02 | 0.17 | 0.17 | 0.10 | 0.06 |
| Marital status | -0.50 | -0.29 | -0.19 | -0.21 | -0.50 | -0.29 | -0.20 | -0.22 |
| Industry type | -0.38 | -0.53 | -0.09 | -0.09 | -0.39 | -0.54 | -0.15 | -0.15 |
| Annual income | 0.02 | 0.02 | 0.03 | 0.02 | 0.01 | 0.02 | 0.03 | 0.02 |
| Business size | 0.05 | 0.01 | -0.02 | -0.06 | -0.07 | -0.11 | -0.14 | -0.17 |
|  |  |  |  |  |  |  |  |  |
| R-squared | 0.06 | 0.40 | 0.64 | 0.67 | 0.07 | 0.39 | 0.58 | 0.59 |
| R-squared change |  | 0.34 | 0.24 | 0.03 |  | 0.32 | 0.19 | 0.01 |
| *N = 80* |  |  |  |  |  |  |  |  |

**Table D.2:**  Moderated hierarchical regressions toward e-tax service continuance intention

| Variables | Perceived usefulness | | | | Perceived ease of use | | | |
| --- | --- | --- | --- | --- | --- | --- | --- | --- |
|  | Model 1 | Model 2 | Model 3 | Model 4 | Model 5 | Model 6 | Model 7 | Model 8 |
| Creativity |  | 1.61*** | 0.76*** | 2.44** |  | 1.47*** | 0.48* | 2.16* |
| Entrepreneurial orientation |  |  | 0.60*** | 0.77*** |  |  | 0.70*** | 0.87*** |
| CA X EO |  |  |  | -0.34 |  |  |  | -0.34 |
| Age | -0.05** | -0.01 | 0.01 | 0.01 | -0.05** | -0.01 | 0.01 | 0.01 |
| Gender | -0.48 | -0.37 | -0.15 | -0.15 | -0.19 | -0.09 | 0.16 | 0.16 |
| Education level | 0.23 | 0.10 | -0.09 | -0.11 | 0.27 | 0.15 | -0.07 | -0.09 |
| Marital status | 0.16 | 0.40 | 0.40 | 0.48 | 0.08 | 0.30 | 0.30 | 0.38 |
| Industry type | 0.86** | 0.77*** | 0.66*** | 0.64*** | 0.62 | 0.53 | 0.41* | 0.39* |
| Annual income | -0.10 | 0.12 | 0.15 | 0.17 | -0.26 | -0.06 | -0.02 | -0.01 |
| Business size | 0.08 | -0.01 | -0.04 | -0.05 | 0.15 | 0.08 | 0.04 | 0.03 |
|  |  |  |  |  |  |  |  |  |
| R-squared | 0.17 | 0.57 | 0.72 | 0.73 | 0.16 | 0.50 | 0.71 | 0.72 |
| R-squared change |  | 0.40 | 0.15 | 0.01 |  | 0.34 | 0.19 | 0.01 |
| *N = 86* |  |  |  |  |  |  |  |  |

**Table D.3:**  Moderated hierarchical regressions toward e- marketplace adoption intention

| Variables | Perceived usefulness | | | | Perceived ease of use | | | |
| --- | --- | --- | --- | --- | --- | --- | --- | --- |
|  | Model 1 | Model 2 | Model 3 | Model 4 | Model 5 | Model 6 | Model 7 | Model 8 |
| Creativity |  | 1.37*** | 0.05 | 0.55 |  | 1.26*** | -0.13 | 0.84 |
| Entrepreneurial orientation |  |  | 0.90*** | 0.93*** |  |  | 0.94*** | 1.01*** |
| CA X EO |  |  |  | -0.10 |  |  |  | -0.20 |
| Age | -0.01 | 0.01 | -0.01 | 0.01 | -0.01 | 0.01 | -0.01 | 0.01 |
| Gender | -0.24 | -0.20 | 0.02 | 0.02 | -0.23 | -0.19 | 0.04 | 0.03 |
| Education level | -0.02 | -0.01 | -0.02 | -0.01 | -0.02 | -0.02 | -0.02 | -0.01 |
| Marital status | -0.28 | -0.07 | -0.03 | -0.02 | -0.16 | 0.04 | 0.08 | 0.09 |
| Industry type | 0.14 | 0.03 | 0.01 | -0.01 | 0.07 | -0.03 | -0.05 | -0.08 |
| Annual income | -0.30 | -0.50 | -0.38* | -0.38* | -0.30 | -0.48 | -0.34 | -0.36 |
| Business size | 0.08 | -0.09 | 0.14 | -0.13 | 0.02 | 0.01 | -0.04 | -0.01 |
|  |  |  |  |  |  |  |  |  |
| R-squared | 0.03 | 0.40 | 0.77 | 0.77 | 0.02 | 0.37 | 0.75 | 0.75 |
| R-squared change |  | 0.37 | 0.37 | 0.00 |  | 0.35 | 0.38 | 0.00 |
| *N = 112* |  |  |  |  |  |  |  |  |

**Table D.4:**  Moderated hierarchical regressions toward e- marketplace continuance intention

| Variables | Perceived usefulness | | | | Perceived ease of use | | | |
| --- | --- | --- | --- | --- | --- | --- | --- | --- |
|  | Model 1 | Model 2 | Model 3 | Model 4 | Model 5 | Model 6 | Model 7 | Model 8 |
| Creativity |  | 1.01*** | 0.26 | 1.56 |  | 0.90*** | 0.30 | 1.07 |
| entrepreneurial orientation |  |  | 0.54*** | 0.68*** |  |  | 0.45*** | 0.54*** |
| CA X EO |  |  |  | -0.27 |  |  |  | -0.16 |
| Age | -0.07*** | -0.05** | -0.03** | -0.03** | -0.06*** | -0.04*** | -0.03 | -0.03 |
| Gender | 0.29 | 0.32 | 0.14 | 0.12 | 0.22 | 0.24 | 0.09 | 0.09 |
| Education level | 0.08 | -0.01 | 0.08 | 0.08 | 0.08 | -0.01 | 0.08 | 0.07 |
| Marital status | -0.37 | -0.23 | 0.04 | 0.10 | -0.19 | -0.07 | 0.16 | 0.20 |
| Industry type | 0.60 | 0.53* | 0.51* | 0.52* | 0.58* | 0.52** | 0.50** | 0.51** |
| Annual income | -0.51 | -0.45 | -0.28 | -0.25 | -0.54* | -0.48* | -0.33 | -0.32 |
| Business size | 0.33 | 0.29 | 0.17 | 0.16 | 0.26 | 0.22 | 0.13 | 0.12 |
|  |  |  |  |  |  |  |  |  |
| R-squared | 0.39 | 0.60 | 0.72 | 0.72 | 0.40 | 0.63 | 0.74 | 0.75 |
| R-squared change |  | 0.21 | 0.12 | 0.00 |  | 0.23 | 0.11 | 0.01 |
| *N = 63* |  |  |  |  |  |  |  |  |

**Notes:** CA X EO = The interaction of creativity and entrepreneurial orientation

Significant level: *** *p*<0.001, ** *p*<0.01, * *p*<0.05

**Figure D:** Moderation effects

| 1. Perceived usefulness for e-tax service adoption intention | 1. Perceived ease of use for e-tax service adoption intention |
| --- | --- |
| 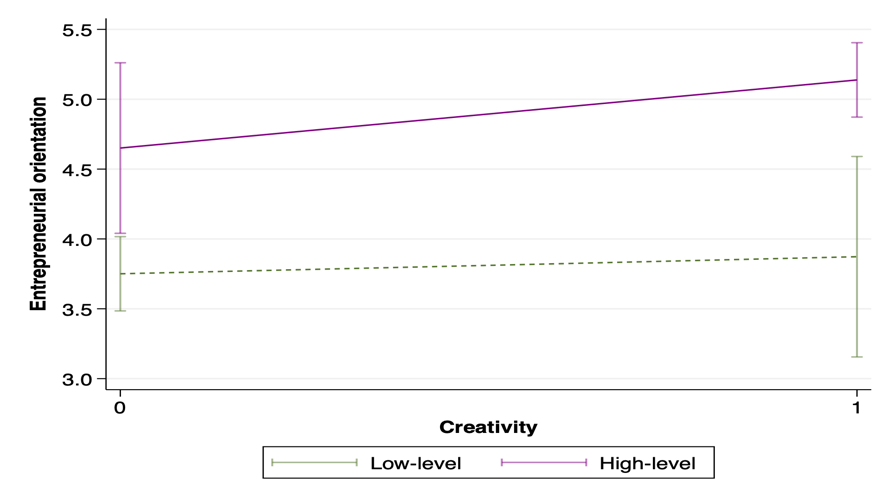 | 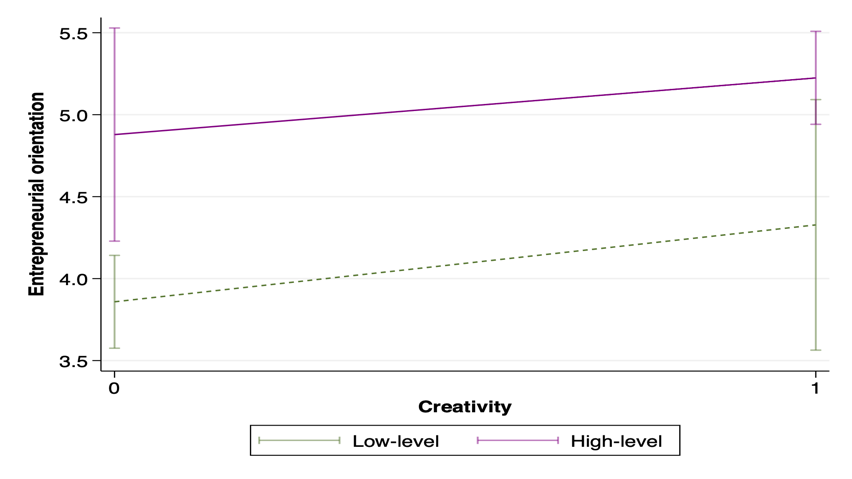 |

| 1. Perceived usefulness for e-tax service continuance intention | 1. Perceived ease of use for e-tax service continuance intention |
| --- | --- |
| 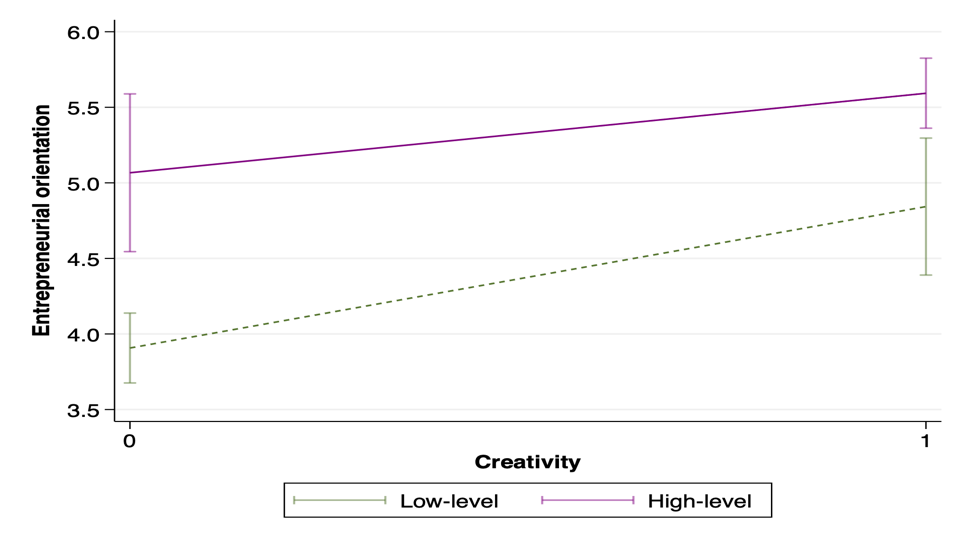 | 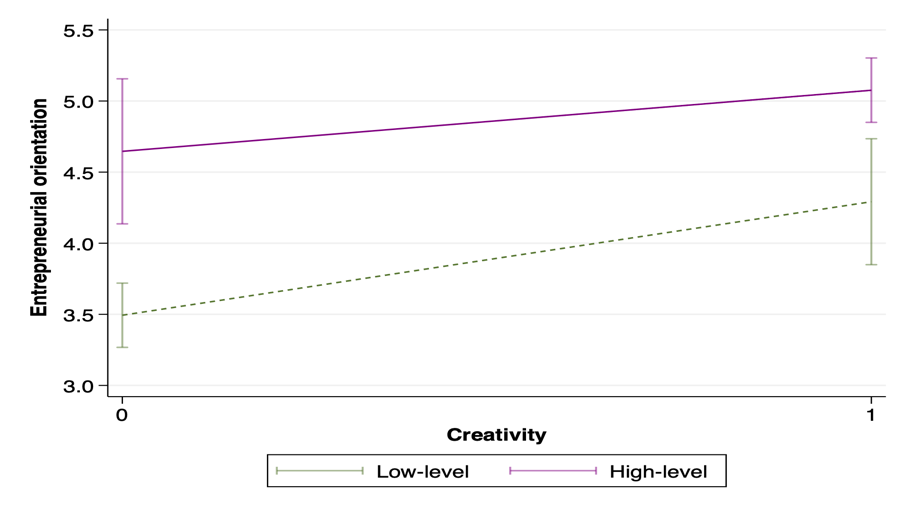 |

| 1. Perceived usefulness for e-marketplace adoption intention | 1. Perceived ease of use for e-marketplace adoption intention |
| --- | --- |
| 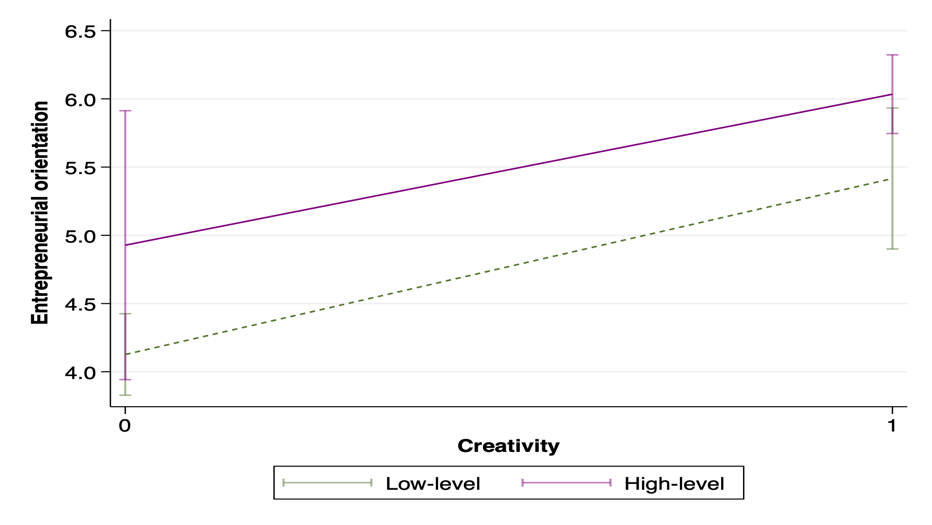 | 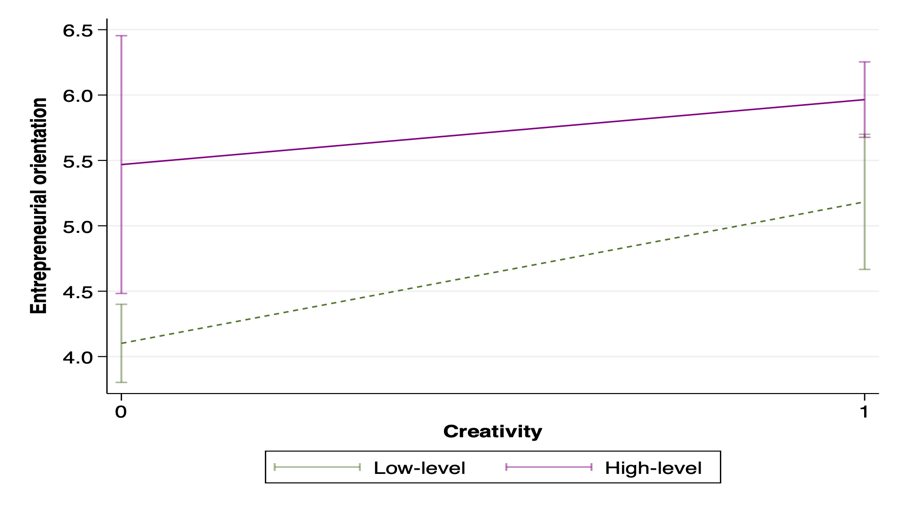 |

| 1. Perceived usefulness for e-marketplace continuance intention | 1. Perceived ease of use for e-marketplace continuance intention |
| --- | --- |
| 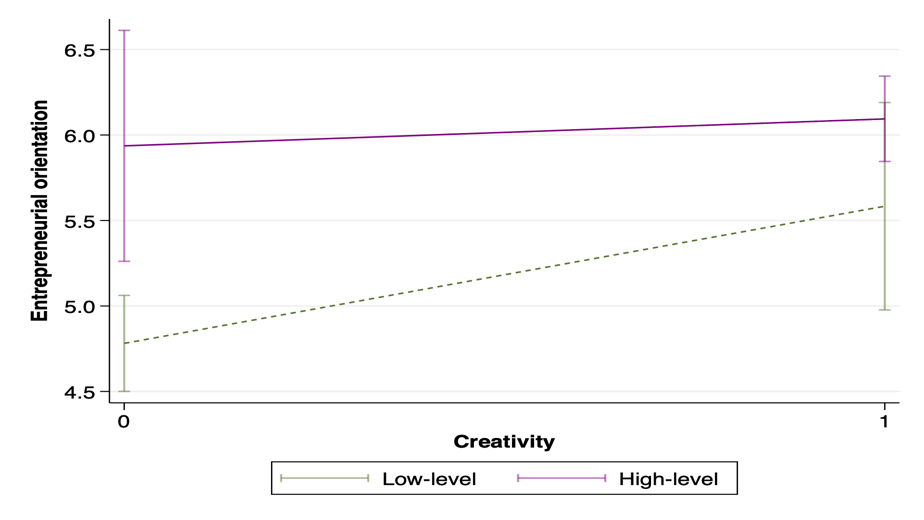 | 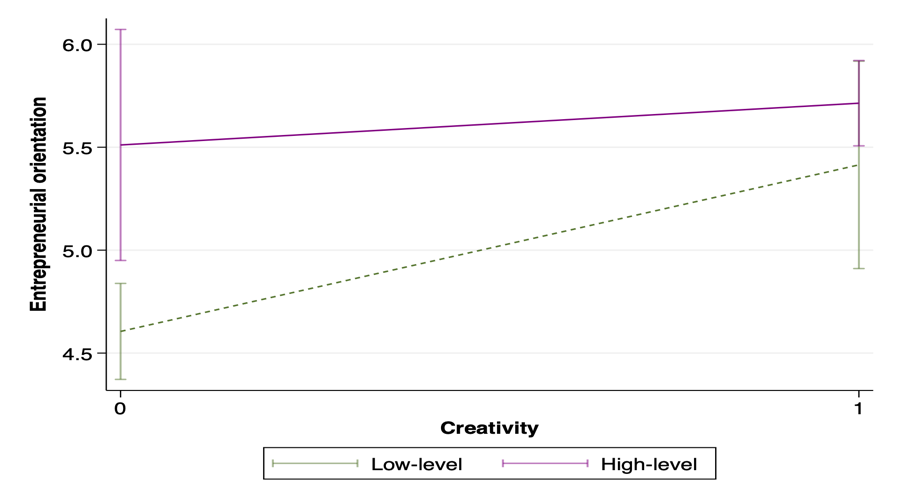 |

**Appendix E.** Measurement for the Main Variables

| Variables | Items of variables |
| --- | --- |
| Creativity | 1. I am a very creative person. 2. I set aside a few minutes each day or week to be creative. 3. I have plenty of ideas. 4. I search for new solutions even when they are not needed. 5. My ideas are often very original. 6. I am sensitive to seeing problems that others do not see. 7. New solutions, also, come into my mind when they are not especially needed. 8. It is easy for me to find proposals for improvement. |
| Entrepreneurial orientation | 1. If an entrepreneurial firm is operationally defined as “one that engages in product-market innovation, undertakes somewhat risky ventures, and is first to come up with ‘proactive’ innovations, beating competitors to the punch,” then my firm is an entrepreneurial firm. 2. My firm characteristically exhibits high levels of risk taking, innovativeness, and proactiveness. 3. My firm often takes calculated risks by pursuing innovative initiatives before potential rivals recognize the opportunities at which our 4. initiatives are targeted. 5. Risk taking, innovativeness, and proactiveness are equally inherent to my firm’s overall business orientation. 6. The innovative initiatives pursued/funded by my firm are often somewhat risky and industry leading (i.e., chosen in advance of other 7. firms’ potentially similar initiatives). 8. My firm concurrently manifests risk taking, innovativeness, and proactiveness. 9. My firm often pre-empts its rivals by being an early leader with innovations whose successful outcomes cannot be assured. 10. In general, my firm is on the cutting edge when it comes to exploiting entrepreneurial opportunities because of our desire and 11. demonstrated ability to embrace novel (and often risky) innovative initiatives ahead of our rivals. |
| Perceived usefulness (DJP Online) | 1. I would find DJP Online useful for my business. 2. Using DJP Online enables me to settle tax administration more quickly. 3. Using DJP Online increase my productivity. 4. If I use DJP Online, I will increase my chances of getting more cost and time effectiveness. |
| Perceived ease of use (DJP Online) | 1. My interaction with DJP Online would be clear and understandable. 2. It would be easy for me to become skillful at using DJP Online. 3. I would find DJP Online is easy to use. 4. Learning to use DJP Online is easy for me. |
| DJP Online adoption intention | 1. I intend to use DJP Online in the near future. 2. I predict I would use DJP Online in the near future. 3. I plan to use DJP Online in the near future. |
| DJP Online continuance intention | 1. I intend to continue using DJP Online rather than discontinue its use. 2. My intentions are to continue using DJP Online than use any alternative means (in person services, by letter, etc.). 3. If I could, I would like to discontinue my use of DJP Online. |
| Perceived usefulness (e-marketplace) | 1. I would find e-marketplace useful in my business. 2. Using e-marketplace enables me to accomplish tasks more quickly. 3. Using e-marketplace increase my productivity. 4. If I use an e-marketplace, I will increase my chances of getting more customer. |
| Perceived ease of use (e-marketplace) | 1. My interaction with the e-marketplace would be clear and understandable. 2. It would be easy for me to become skillful at using e-marketplace. 3. I would find e-marketplace is easy to operate. 4. Learning to operate an e-marketplace is easy for me. |
| E-marketplace adoption intention | 1. I intend to use e-marketplace in the near future. 2. I predict I would use e-marketplace in the near future. 3. I plan to use e-marketplace in the near future. |
| E-marketplace continuance intention | 1. I intend to continue using e-marketplace rather than discontinue its use. 2. My intentions are to continue using e-marketplace than use any alternative means (traditional shop). 3. If I could, I would like to discontinue my use of e-marketplace. |

**Appendix F.** Research Questionnaire

| **RESEARCH QUESTIONNAIRE** |
| --- |
| Dear entrepreneurs,  I am a doctoral student of the XXX. My research topic will be analyzing the Entrepreneurs’ Behavior on Information Technology (IT) Adoption, specifically e-marketplace and DJP Online.  I reach to invite you to participate in the survey regarding the entrepreneurs’ opinions in Indonesia. This research is conducted to fulfil one of the requirements for my graduation. You only need to spend 10 minutes to fill all questions. Your participation is voluntarily and without any further consequences whatsoever. You are able to quit any time if you do not feel comfortable regarding this survey.  The data collected through this questionnaire is solely for academic purposes, and the identity and answers given is guaranteed to be confidential. Therefore, please be kind to answer all the questions.  Best regards |

| **RESPONDENTS DATA** | | | | |  |  | | |
| --- | --- | --- | --- | --- | --- | --- | --- | --- |
| 1. Age (years old) | ………………………………………. | | | |  | 1. Average annual revenue (rupiahs) | 1 | < 250.000.000 |
| 1. Gender | 1 | Male | 2 | Female |  |  | 2 | 250.000.000 ≤ X < 500.000.000 |
| 1. Educational background | 1 | SD | | |  |  | 3 | 500.000.000 ≤ X < 750.000.000 |
|  | 2 | SMP | | |  |  | 4 | 750.000.000 ≤ X < 1.000.000.000 |
|  | 3 | SMA | | |  |  | 5 | 1.000.000.000 ≤ X < 1.250.000.000 |
|  | 4 | D3/S1 | | |  |  | 6 | 1.250.000.000 ≤ X < 1.500.000.000 |
|  | 5 | S2/S3 | | |  |  | 7 | 1.500.000.000 ≤ X < 1.750.000.000 |
| 1. Marital status | 1 | Married | | |  |  | 8 | 1.750.000.000 ≤ X < 2.000.000.000 |
|  | 2 | Not married | | |  |  | 9 | ³ 2.000.000.000 |
| 1. City of residence | ­­­……………………………………… | | | |  | 1. Employee number | 1 | Self-employed |
| 1. Business category | 1 | Trade | | |  |  | 2 | 1 ≤ X ≤ 5 |
|  | 2 | Non-Trade | | |  |  | 3 | 6 ≤ X ≤ 10 |
|  |  |  |  |  |  |  | 4 | 11 ≤ X ≤ 15 |
|  | | | | |  |  | 5 | 16 ≤ X ≤ 20 |
|  |  |  |  |  |  |  | 6 | > 20 |

| STATEMENTS | STRONGLY DISAGREE | DISAGREE | SOMEWHAT DISAGREE | NEUTRAL | SOMEWHAT AGREE | AGREE | STRONGLY AGREE |
| --- | --- | --- | --- | --- | --- | --- | --- |
| 1. Please give us your opinion related to the following aspects of the CREATIVITY | | | | | | | |
| I am a very creative person |  |  |  |  |  |  |  |
| I set aside a few minutes each day or week to be creative |  |  |  |  |  |  |  |
| I have plenty of ideas |  |  |  |  |  |  |  |
| I search for new solutions even when they are not needed |  |  |  |  |  |  |  |
| My ideas are often very original |  |  |  |  |  |  |  |
| I am sensitive to seeing problems that others do not see |  |  |  |  |  |  |  |
| New solutions, also, come into my mind when they are not especially needed |  |  |  |  |  |  |  |
| It is easy for me to find proposals for improvement |  |  |  |  |  |  |  |

| STATEMENTS | STRONGLY DISAGREE | DISAGREE | SOMEWHAT DISAGREE | NEUTRAL | SOMEWHAT AGREE | AGREE | STRONGLY AGREE |
| --- | --- | --- | --- | --- | --- | --- | --- |
| 1. Please give us your opinion related to the following aspects of the ENTREPRENEURIAL ORIENTATION | | | | | | | |
| If an entrepreneurial firm is operationally defined as “one that engages in product-market innovation, undertakes somewhat risky ventures, and is first to come up with ‘proactive’ innovations, beating competitors to the punch,” then my firm is an entrepreneurial firm. |  |  |  |  |  |  |  |
| My firm characteristically exhibits high levels of risk taking, innovativeness, and proactiveness. |  |  |  |  |  |  |  |
| My firm often takes calculated risks by pursuing innovative initiatives before potential rivals recognize the opportunities at which our  initiatives are targeted. |  |  |  |  |  |  |  |
| Risk taking, innovativeness, and proactiveness are equally inherent to my firm’s overall business orientation. |  |  |  |  |  |  |  |
| The innovative initiatives pursued/funded by my firm are often somewhat risky and industry leading (i.e., chosen in advance of other  firms’ potentially similar initiatives). |  |  |  |  |  |  |  |
| My firm concurrently manifests risk taking, innovativeness, and proactiveness. |  |  |  |  |  |  |  |
| My firm often pre-empts its rivals by being an early leader with innovations whose successful outcomes cannot be assured. |  |  |  |  |  |  |  |
| In general, my firm is on the cutting edge when it comes to exploiting entrepreneurial opportunities because of our desire and  demonstrated ability to embrace novel (and often risky) innovative initiatives ahead of our rivals. |  |  |  |  |  |  |  |

| STATEMENTS | STRONGLY DISAGREE | DISAGREE | SOMEWHAT DISAGREE | NEUTRAL | SOMEWHAT AGREE | AGREE | STRONGLY AGREE |
| --- | --- | --- | --- | --- | --- | --- | --- |
| 1. Please give us your opinion related to the following aspects of the PERCEIVED USEFULNESS related to DJP ONLINE | | | | | | | |
| I would find DJP Online useful for my business |  |  |  |  |  |  |  |
| Using DJP Online enables me to settle tax administration more quickly |  |  |  |  |  |  |  |
| Using DJP Online increase my productivity |  |  |  |  |  |  |  |
| If I use DJP Online, I will increase my chances of getting more cost and time effectiveness |  |  |  |  |  |  |  |

| STATEMENTS | STRONGLY DISAGREE | DISAGREE | SOMEWHAT DISAGREE | NEUTRAL | SOMEWHAT AGREE | AGREE | STRONGLY AGREE |
| --- | --- | --- | --- | --- | --- | --- | --- |
| 1. Please give us your opinion related to the following aspects of the PERCEIVED EASE OF USE related to DJP ONLINE | | | | | | | |
| My interaction with DJP Online would be clear and understandable |  |  |  |  |  |  |  |
| It would be easy for me to become skilful at using DJP Online |  |  |  |  |  |  |  |
| I would find DJP Online is easy to use |  |  |  |  |  |  |  |
| Learning to use DJP Online is easy for me |  |  |  |  |  |  |  |

| STATEMENTS | STRONGLY DISAGREE | DISAGREE | SOMEWHAT DISAGREE | NEUTRAL | SOMEWHAT AGREE | AGREE | STRONGLY AGREE |
| --- | --- | --- | --- | --- | --- | --- | --- |
| 1. Please give us your opinion related to the following aspects of the DJP ONLINE ADOPTION | | | | | | | |
| Using DJP Online is a good idea |  |  |  |  |  |  |  |
| DJP Online makes tax services more interesting |  |  |  |  |  |  |  |
| Settling tax services with DJP Online is fun |  |  |  |  |  |  |  |
| I like settling tax services with DJP Online |  |  |  |  |  |  |  |

| STATEMENTS | STRONGLY DISAGREE | DISAGREE | SOMEWHAT DISAGREE | NEUTRAL | SOMEWHAT AGREE | AGREE | STRONGLY AGREE |
| --- | --- | --- | --- | --- | --- | --- | --- |
| 1. Please give us your opinion related to the following aspects of the PERCEIVED USEFULNESS related to E-MARKETPLACE | | | | | | | |
| I would find e-marketplace useful in my business |  |  |  |  |  |  |  |
| Using e-marketplace enables me to accomplish tasks more quickly |  |  |  |  |  |  |  |
| Using e-marketplace increase my productivity |  |  |  |  |  |  |  |
| If I use an e-marketplace, I will increase my chances of getting more customer |  |  |  |  |  |  |  |

| STATEMENTS | STRONGLY DISAGREE | DISAGREE | SOMEWHAT DISAGREE | NEUTRAL | SOMEWHAT AGREE | AGREE | STRONGLY AGREE |
| --- | --- | --- | --- | --- | --- | --- | --- |
| 1. Please give us your opinion related to the following aspects of the PERCEIVED EASE OF USE related to E-MARKETPLACE | | | | | | | |
| My interaction with the e-marketplace would be clear and understandable |  |  |  |  |  |  |  |
| It would be easy for me to become skilful at using e-marketplace |  |  |  |  |  |  |  |
| I would find e-marketplace is easy to operate |  |  |  |  |  |  |  |
| Learning to operate an e-marketplace is easy for me |  |  |  |  |  |  |  |

| STATEMENTS | STRONGLY DISAGREE | DISAGREE | SOMEWHAT DISAGREE | NEUTRAL | SOMEWHAT AGREE | AGREE | STRONGLY AGREE |
| --- | --- | --- | --- | --- | --- | --- | --- |
| 1. Please give us your opinion related to the following aspects of the E-MARKETPLACE ADOPTION | | | | | | | |
| Using e-marketplace is a good idea |  |  |  |  |  |  |  |
| E-marketplace makes work more interesting |  |  |  |  |  |  |  |
| Working with e-marketplace is fun |  |  |  |  |  |  |  |
| I like working with e-marketplace |  |  |  |  |  |  |  |

| **EXPERIENCES** | | |  | | |
| --- | --- | --- | --- | --- | --- |
| 1. Has your venture ever used an e-marketplace? |  | Yes | 1. Has your venture ever used DJP Online? |  | Yes |
|  |  | No |  |  | No |
| If you answer to Yes, approximately how long have you use the e-marketplace (years)? |  | ≤ 1 | If you answer to Yes, approximately how long have you use DJP Online (years)? |  | ≤ 1 |
|  |  | 1 ≤ X < 2 |  |  | 1 ≤ X < 2 |
|  |  | 2 ≤ X < 3 |  |  | 2 ≤ X < 3 |
|  |  | 3 ≤ X < 4 |  |  | 3 ≤ X < 4 |
|  |  | 4 ≤ X < 5 |  |  | 4 ≤ X < 5 |
|  |  | ≥ 5 |  |  | ≥ 5 |

**If you answer to ‘Yes’, please proceed to questions 23 and 24**

**If you answer to ‘No’, please proceed to questions 25 and 26**

| STATEMENTS | STRONGLY DISAGREE | DISAGREE | SOMEWHAT DISAGREE | NEUTRAL | SOMEWHAT AGREE | AGREE | STRONGLY AGREE |
| --- | --- | --- | --- | --- | --- | --- | --- |
| 1. Please give us your opinion related to the following aspects of the USE CONTINUANCE INTENTION related to DJP ONLINE | | | | | | | |
| I intend to continue using DJP Online rather than discontinue its use |  |  |  |  |  |  |  |
| My intentions are to continue using DJP Online than use any alternative means (in person services, by letter, etc.) |  |  |  |  |  |  |  |
| If I could, I would like to discontinue my use of DJP Online |  |  |  |  |  |  |  |

| STATEMENTS | STRONGLY DISAGREE | DISAGREE | SOMEWHAT DISAGREE | NEUTRAL | SOMEWHAT AGREE | AGREE | STRONGLY AGREE |
| --- | --- | --- | --- | --- | --- | --- | --- |
| 1. Please give us your opinion related to the following aspects of the USE CONTINUANCE INTENTION related to E-MARKETPLACE | | | | | | | |
| I intend to continue using e-marketplace rather than discontinue its use |  |  |  |  |  |  |  |
| My intentions are to continue using e-marketplace than use any alternative means (traditional shop) |  |  |  |  |  |  |  |
| If I could, I would like to discontinue my use of e-marketplace |  |  |  |  |  |  |  |
